# JMJD6 Promotes Colon Carcinogenesis through Negative Regulation of p53 by Hydroxylation

**DOI:** 10.1371/journal.pbio.1001819

**Published:** 2014-03-25

**Authors:** Feng Wang, Lin He, Peiwei Huangyang, Jing Liang, Wenzhe Si, Ruorong Yan, Xiao Han, Shumeng Liu, Bin Gui, Wanjin Li, Di Miao, Chao Jing, Zhihua Liu, Fei Pei, Luyang Sun, Yongfeng Shang

**Affiliations:** 1 Key Laboratory of Carcinogenesis and Translational Research (Ministry of Education), Department of Biochemistry and Molecular Biology, Peking University Health Science Center, Beijing, China; 2 2011 Collaborative Innovation Center of Tianjin for Medical Epigenetics, Tianjin Key Laboratory of Medical Epigenetics, Department of Biochemistry and Molecular Biology, Tianjin Medical University, Tianjin, China; 3 Proteomics Facility, School of Life Sciences, Tsinghua University, Beijing, China; 4 State Key Laboratory of Molecular Oncology, The Cancer Institute, Chinese Academy of Medical Sciences, Peking Union Medical College, Beijing, China; 5 Department of Pathology, Peking University Health Science Center, Beijing, China; Mount Sinai School of Medicine, United States of America

## Abstract

Jumonji domain-containing 6 (JMJD6) is a member of the Jumonji C domain-containing family of proteins. Compared to other members of the family, the cellular activity of JMJD6 is still not clearly defined and its biological function is still largely unexplored. Here we report that JMJD6 is physically associated with the tumor suppressor p53. We demonstrated that JMJD6 acts as an α-ketoglutarate– and Fe(II)-dependent lysyl hydroxylase to catalyze p53 hydroxylation. We found that p53 indeed exists as a hydroxylated protein *in vivo* and that the hydroxylation occurs mainly on lysine 382 of p53. We showed that JMJD6 antagonizes p53 acetylation, promotes the association of p53 with its negative regulator MDMX, and represses transcriptional activity of p53. Depletion of JMJD6 enhances p53 transcriptional activity, arrests cells in the G_1_ phase, promotes cell apoptosis, and sensitizes cells to DNA damaging agent-induced cell death. Importantly, knockdown of JMJD6 represses p53-dependent colon cell proliferation and tumorigenesis *in vivo*, and significantly, the expression of JMJD6 is markedly up-regulated in various types of human cancer especially in colon cancer, and high nuclear JMJD6 protein is strongly correlated with aggressive clinical behaviors of colon adenocarcinomas. Our results reveal a novel posttranslational modification for p53 and support the pursuit of JMJD6 as a potential biomarker for colon cancer aggressiveness and a potential target for colon cancer intervention.

## Abstract 2

p53 hydroxylation by JMJD6 represents a novel post-translational modification for p53. JMJD6-mediated hydroxylation regulates p53's transcriptional activity and the p53-dependent control of colon cancer.

## Author Summary

JMJD6 belongs to the Jumonji C domain-containing family of proteins. The majority of this family are histone demethylases implicated in chromatin-associated events, but there have also been some reports of lysyl hydroxylase activity for JMJD6. Here we report a new posttranslational modification for the tumor suppressor protein p53 that is mediated by JMJD6. Via a physical associations with p53, JMJD6 catalyzes the hydroxylation of p53, thereby repressing its transcriptional activity. Depletion of JMJD6 promotes cell apoptosis, arrests cells in the G1 phase, sensitizes cells to DNA damaging agent-induced cell death, and represses p53-dependent colon cell proliferation and tumorigenesis. Significantly, the expression of JMJD6 is markedly up-regulated in various types of human cancer especially in colon cancer, and high nuclear JMJD6 protein is strongly correlated with aggressive clinical behaviors of colon adenocarcinomas. Our results support the pursuit of JMJD6 as a potential biomarker for colon cancer aggressiveness and a potential target for colon cancer intervention.

## Introduction

Jumonji domain-containing 6 (JMJD6) is a member of the Jumonji C domain-containing family of proteins. The majority of proteins in this family have been characterized as histone demethylases that are implicated in chromatin remodeling. Interestingly, JMJD6 was first identified as a phosphatidylserine receptor on cell membrane functioning in phagocytosis of apoptotic cells [1]–[3]. Subsequently, it was recognized that JMJD6 possesses catalytic activity as dioxygenase in the nucleus. However, JMJD6 has been described as either a histone demethylase to remove the methyl moieties on histone H3 at arginine 2 (H3R2) and histone H4 at arginine 3 (H4R3) [4] or as a lysyl hydroxylase to target U2AF65 [5], a protein associated with RNA splicing. These observations clearly indicate that the biological function of JMJD6 might be multidimensional. Given current understanding of Jumonji C domain-containing proteins in controlling a wide range of biological functions, the importance and multitude of the cellular activity of JMJD6 is expected.

The p53 protein is important in many aspects of cell biology, including cell cycle control and regulation of apoptosis, metabolism, fertility, differentiation, and cell reprogramming [6]–[8]. It is described as “the guardian of the genome” and believed to coordinate several preeminent signaling pathways that are important to genome integrity and cell survival [8]–[14]. At the cellular level, p53 is able to prevent cells from entering or progressing through the cell cycle under various adverse conditions, and at the molecular level, p53 rapidly accumulates in response to genotoxic stresses and acts as a sequence-specific transcription factor to activate the transcription of an array of downstream genes [11],[15]. The antitumorigenic effects of p53 are thus mediated by its target gene products that govern various cellular activities including cell cycle arrest, apoptosis, and cellular senescence [8]. As inactivation or activation of p53 sets up life or death decisions, sophisticated yet elaborate regulatory mechanisms have evolved to finely tune the protein level of p53.

Under normal cell growth conditions, the level of p53 protein is kept low through regulation of its stability by a number of negative regulators. Current literature including studies from our own lab indicates that p53 degradation is mediated by several ubiquitin ligases, including MDM2 [16]–[20], Pirh2 [21], COP1 [22], ARF-BP1 [23], and JFK [24],[25], and interestingly, there exist autoregulatory negative feedback loops between p53 and each of MDM2, Pirh2, COP1, and JFK. Interestingly, however, a recent study using quantitative time-lapse microscopy of individual human cells found that proliferating cells exhibit spontaneous pulses of p53 that are correlated with cell cycle events [26]. It was reported that fluctuations in p53 protein level reflect the shift of asynchronous, spontaneous pulses to a series of regular, high-frequency, and synchronized oscillations from unstressed cells to stressed cells [26], suggesting that the amount of p53 is not the only primary reason for p53 activation and underscoring the importance of posttranslational modifications in p53 regulation.

Posttranslational modifications of p53 have been implicated in the regulation of the stability and activity of p53 for a long time [6],[27],[28]. p53 protein can be modified through phosphorylation by several kinases including ataxia telangiectasia mutated (ATM), ATM- and Rad3-related (ATR), DNA-dependent protein kinase (DNA-PK), and checkpoint kinase 1/2 (CHK1/2) [29]–[34]. Phosphorylation of p53 results in inhibition of its interaction with MDM2 [35] or in retention of p53 protein in the nucleus [36] and is thus often associated with a positive effect on the p53 pathway. p53 can also be modified by p300/CBP and Tip60 by virtue of their acetyltransferase activity [37],[38]. Analogously, acetylation is associated with positive p53 regulation owing to enhanced sequence-specific DNA binding capacity of acetylated p53 protein [37] or disrupted interaction of p53 with MDMX, a primary p53 corepressor [39]. However, in contrast to the extensive characterization of instances in which phosphorylation and acetylation positively influence p53 activity, the information about the modifications that might be associated with negative p53 regulation is relatively limited. This issue is of particular importance as a balance between activation and repression is essential for tightly regulated transcriptional activity of p53. Although p53 has been found to be methylated by SMYD2 and SET8/PR-SET7/KMT5A at lysine 370 and 382, respectively, resulting in negative regulation of p53 activity [40],[41], there is still a wide gap in what is known about positive versus negative regulation of p53 in the context of posttranslational modifications.

Here we report that the p53 tumor suppressor also exists as a hydroxylated protein *in vivo* and that JMJD6 acts as an Fe(II)- and α-ketoglutarate–dependent lysyl hydroxylase to catalyze p53 hydroxylation. We demonstrated that p53 hydroxylation inhibits its acetylation and represses its transcriptional activity, and that depletion of JMJD6 enhances p53 transcriptional activity, arrests cells in the G_1_ phase, promotes cell apoptosis, and sensitizes cells to DNA damaging agent-induced cell death. We showed that knockdown of JMJD6 represses p53-dependent cell proliferation and tumorigenesis *in vivo*. We found that the JMJD6 is overexpressed in various human cancers especially in colon cancer, and that high nuclear JMJD6 protein is associated with aggressive clinical behaviors of colon adenocarcinomas.

## Results

### JMJD6 Is Physically Associated with p53 *in Vivo* and *in Vitro*

**Figure 1:**
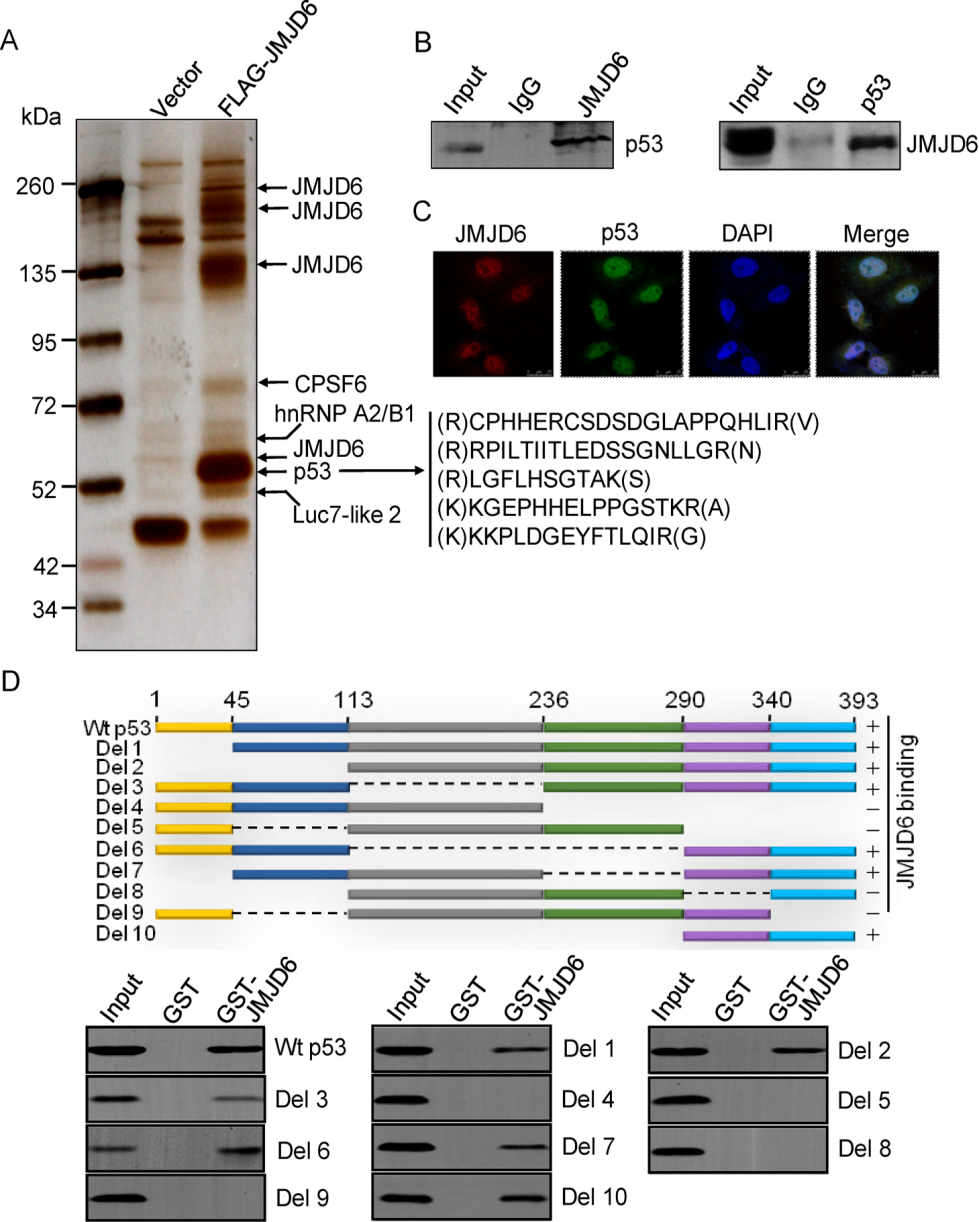
JMJD6 is physically associated with p53 *in vivo* and *in vitro*. (A) Cellular extracts from HCT116 cells stably expressing vector or FLAG-JMJD6 were immunopurified with anti-FLAG affinity columns and eluted with FLAG peptide. The eluates were resolved by SDS-PAGE and silver-stained. The proteins bands were retrieved and analyzed by mass spectrometry. (B) HCT116 cell lysates were immunoprecipitated with antibodies against JMJD6 followed by immunoblotting with antibodies against p53 (FL-393), or they were immunoprecipitated with antibodies against p53 (FL-393) followed by immunoblotting with antibodies against JMJD6. (C) Immunofluorescence-stained endogenous JMJD6 (red) and p53 (green) were visualized by confocal microscopy. DAPI staining was included to visualize the cell nucleus (blue). Scale bar, 25 µm. (D) Mapping the domain of p53 that is required for its interaction with JMJD6. GST pull-down experiments were performed with GST-fused JMJD6 and *in vitro* transcribed/translated Myc-tagged full-length p53 or deletions of p53.

Epigenetic regulation of gene transcription is one of the primary research focuses in our laboratory [42]–[45]. In order to further explore the cellular functions of JMJD6, we employed affinity purification and mass spectrometry to identify the proteins that are potentially associated with JMJD6. In these experiments, FLAG-tagged JMJD6 (FLAG-JMJD6) was stably expressed in human colon carcinoma HCT116 cells. Cellular extracts were prepared and subjected to affinity purification using anti-FLAG affinity columns. After extensive washing, the bound proteins were eluted with excess FLAG peptides, resolved on SDS-PAGE, and then visualized by silver staining. The protein bands on the gel were retrieved and analyzed by mass spectrometry. The results indicated that JMJD6 was co-purified with several proteins including cleavage and polyadenylation specific factor 6 (CPSF6), heterogeneous nuclear ribonucleoprotein A2/B1 (hnRNP), putative RNA-binding protein Luc7-like 2, and JMJD6 itself, which was also reported to form homo-oligomer (Figure 1A and Table S1) [46],[47]. Interestingly, five matching peptides from the tumor suppressor protein p53 were also identified in the JMJD6-containing protein complex (Figure 1A), suggesting that JMJD6 is associated with p53 *in vivo*.

In order to confirm the *in vivo* association between JMJD6 and p53, HCT116 cell lysates were prepared and co-immunoprecipitation assays were performed with antibodies against JMJD6 followed by immunoblotting with antibodies against p53. The results showed that p53 was efficiently co-immunoprecipitated with JMJD6 (Figure 1B). Reciprocal immunoprecipitation with anti-p53 and immunoblottings with anti-JMJD6 also indicated that JMJD6 interacts with p53 *in vivo* (Figure 1B). In addition, confocal microscopy of the subcellular localization of immunofluorescence-stained endogenous JMJD6 and p53 in HCT116 cells showed that JMJD6 protein was colocalized with p53 in the nucleus (Figure 1C).

To investigate the molecular detail involved in the interaction of p53 with JMJD6, GST pull-down experiments were performed with bacterially expressed- and glutathione S-transferase (GST)–fused JMJD6 (GST-JMJD6) and *in vitro* transcribed/translated Myc-tagged full-length or deletion mutants of p53. These experiments showed that p53 could interact with JMJD6 *in vitro* and the C-terminal fragment of p53 (from residues 290 to 393) is required for p53 binding to JMJD6 (Figure 1D). Collectively, these experiments support our observation that JMJD6 is physically associated with p53 *in vivo*.

### JMJD6 Hydroxylates p53 *in Vivo* and *in Vitro*

**Figure 2:**
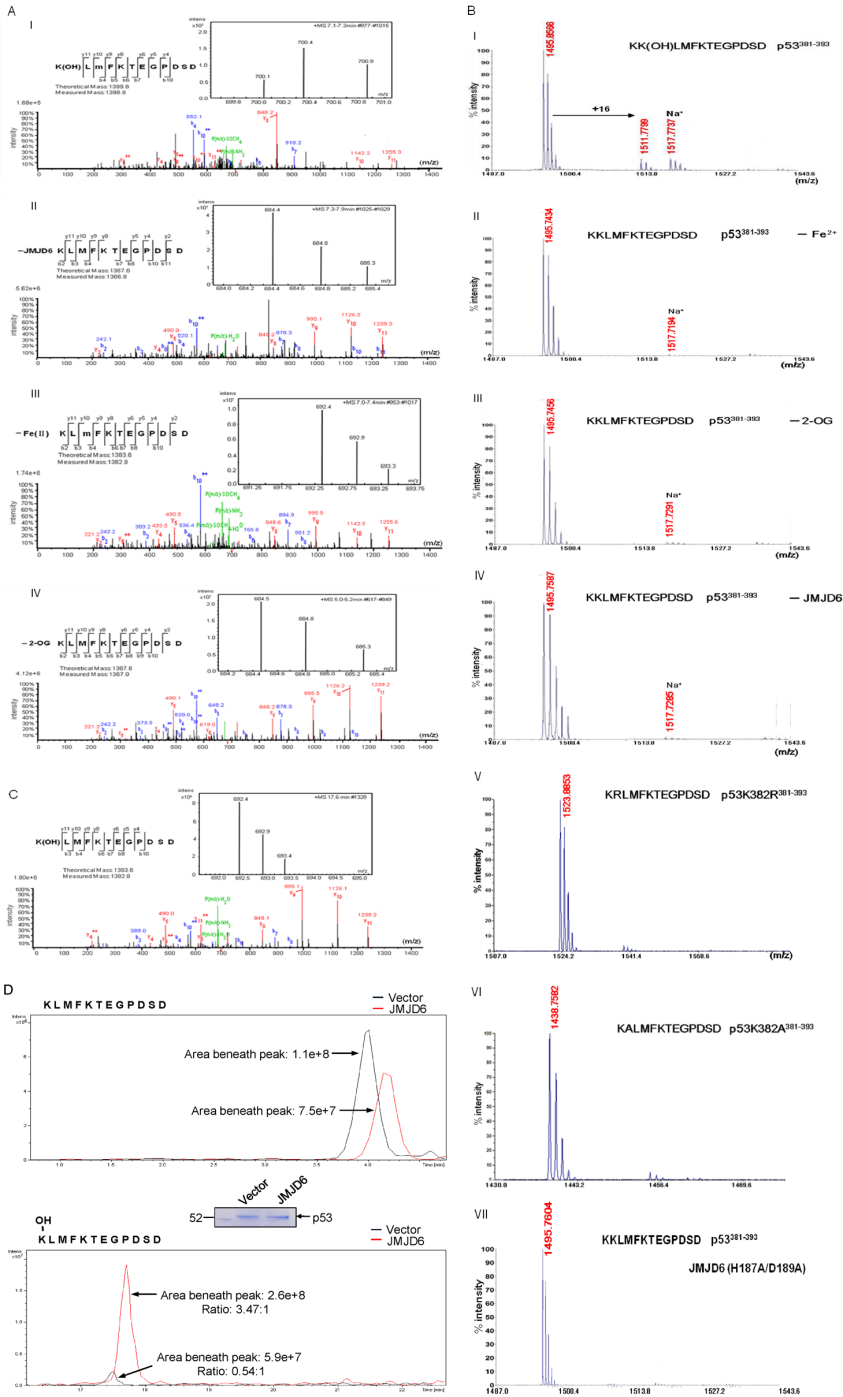
JMJD6 hydroxylates p53 *in vivo* and *in vitro*. (A) Recombinant p53 was incubated with or without recombinant JMJD6 in the presence or absence of α-ketoglutarate (2-OG) and Fe(II). The mixture was then separated on SDS-PAGE, and the band corresponding to the molecular weight of p53 was excised and digested with trypsin and analyzed by LC-MS/MS. Inserts show the doubly charged peptide precursor ions that were fragmented. The relevant ion fragments are labeled, and the corresponding peptide positions are illustrated. K, lysine; K(OH), hydroxylated lysine; the expected increase in mass by hydroxylation modification is 16 Dalton. M, methionine; m, randomly oxidized methionine, which results in a +16 Dalton shift in mass. (I) Experimental group with 2-OG, Fe(II), and JMJD6; (II) negative control group without JMJD6; (III) negative control group without Fe(II); (IV) negative control group without 2-OG. (B) Wild-type JMJD6 hydroxylates p53^381–393^ at K382 of p53 in the presence of 2-OG and Fe(II) *in vitro*. The peptides corresponding to amino acids 381–393 of p53 (wild-type p53, p53K382R, or p53K382A) were incubated with or without recombinant JMJD6 or JMJD6(H187A/D189A) in the presence or absence of 2-OG and Fe(II) for 2 h at 37°C. The mixture was then analyzed by MALDI/TOF. (C) Hydroxylation of p53 at K382 *in vivo*. Lysates from HCT116 cells were immunoprecipitated with anti-p53 monoclonal antibody-conjugated agarose. Bound proteins were eluted with p53 peptide, separated on SDS-PAGE, and analyzed by LC-MS/MS. Inserts show the doubly charged peptide precursor ions that were fragmented. The relevant ion fragments are labeled and the corresponding peptide positions are illustrated. Analysis by LC-MS/MS revealed the presence of modified p53^382–393^ peptide (M+2H)^2+^ containing hydroxylation of K382. (D) Extracted ion chromatogram (XIC) of nonmodified (upper panel) and hydroxylated p53K382 (lower panel) extracted from vector (black) or JMJD6 (red) transfected HCT116 cells.

As stated above, JMJD6 has been reported to act as either a histone demethylase catalyzing the removal of the methyl groups on H3R2 and H4R3 [4] or a lysyl hydroxylase responsible for the addition of hydroxyl groups on nonhistone protein U2AF65 [5]. In order to investigate the biological significance of the physical interaction between JMJD6 with p53, we first incubated bacterially purified GST-p53 with GST-JMJD6 in the presence or absence of Fe(II) and α-ketoglutarate (2-OG) for 2 h at 37°C. The reaction mixture was then resolved on SDS-PAGE and stained with Commassie brilliant blue (CBB). The protein bands on the gels were retrieved and analyzed by liquid chromatography-tandem mass spectrometry (LC-MS/MS). We detected a +16-Dalton mass shift for lysine (K) 382 of the p53 protein, but only when p53 was incubated together with JMJD6 and when Fe(II) and 2-OG were included in the reaction (Figure 2A, I and Table S2A); no hydroxylation of p53 was detected in the reaction mixtures that were without JMJD6, Fe(II), or 2-OG (Figure 2A, II–IV and Table S2B–D). These results support an argument that JMJD6 acts as an 2-OG– and Fe(II)-dependent lysyl hydroxylase to catalyze p53 hydroxylation *in vitro*.

To gain further support of JMJD6-catalyzed p53 hydroxylation, we generated, by site-directed mutagenesis, a catalytically inactive JMJD6 mutant, JMJD6(H187A/D189A); mutation of these two residues is predicted to interrupt the Fe(II) binding of JMJD6 and thus to abolish its hydroxylation activity [4]. Peptides corresponding to amino acid residue 381–393 of p53 including wild-type p53K382 and p53K382R or p53K382A mutants were synthesized and incubated with recombinant GST-JMJD6 or GST-JMJD6(H187A/D189A) in the presence or absence of Fe(II) or 2-OG for 2 h at 37°C. The reaction was then terminated and the materials in the mixture were analyzed by matrix-assisted laser desorption/ionization time-of-flight (MALDI-TOF). We detected, when the peptides were incubated with GST-JMJD6, a +16-Dalton mass shift in wild-type K382-containing p53^381–393^ peptide (Figure 2B, I and Figure S1A), but only when Fe(II) and 2-OG were included in the reactions (Figure 2B, II–IV). No hydroxylation of p53 was detected in K382R- or K382A-containing p53^381–393^ peptide (Figure 2B, V–VI and Figure S1B,C). However, when the peptides were incubated with JMJD6(H187A/D189A), no mass shift was detected, even in wild-type K382-containing p53^381–393^ peptide (Figure 2B, VII and Figure S1D). These experiments support our argument that JMJD6 catalyzes p53 hydroxylation *in vitro*.

Although p53 has been shown to be modified by a variety of enzymatic reactions, hydroxylation of this protein has not been reported before. In order to confirm that p53 is hydroxylated *in vivo*, LC-MS/MS analysis of endogenous p53 that was immunoprecipitated with anti-p53 from volume-prepared HCT116 nuclear extracts revealed that the endogenous p53 is indeed hydroxylated, and the hydroxylation occurs on K382 of p53 (Figure 2C and Table S3). In addition, overexpression of JMJD6 in HCT116 cells resulted in an increase in the amount of K382-hydrxoylated p53 about 6-fold (Figure 2D). These experiments support our arguments that p53 is hydroxylated *in vivo* and that the hydroxylation is catalyzed by JMJD6.

### Negative Regulation of p53 Transcriptional Activity by JMJD6

**Figure 3:**
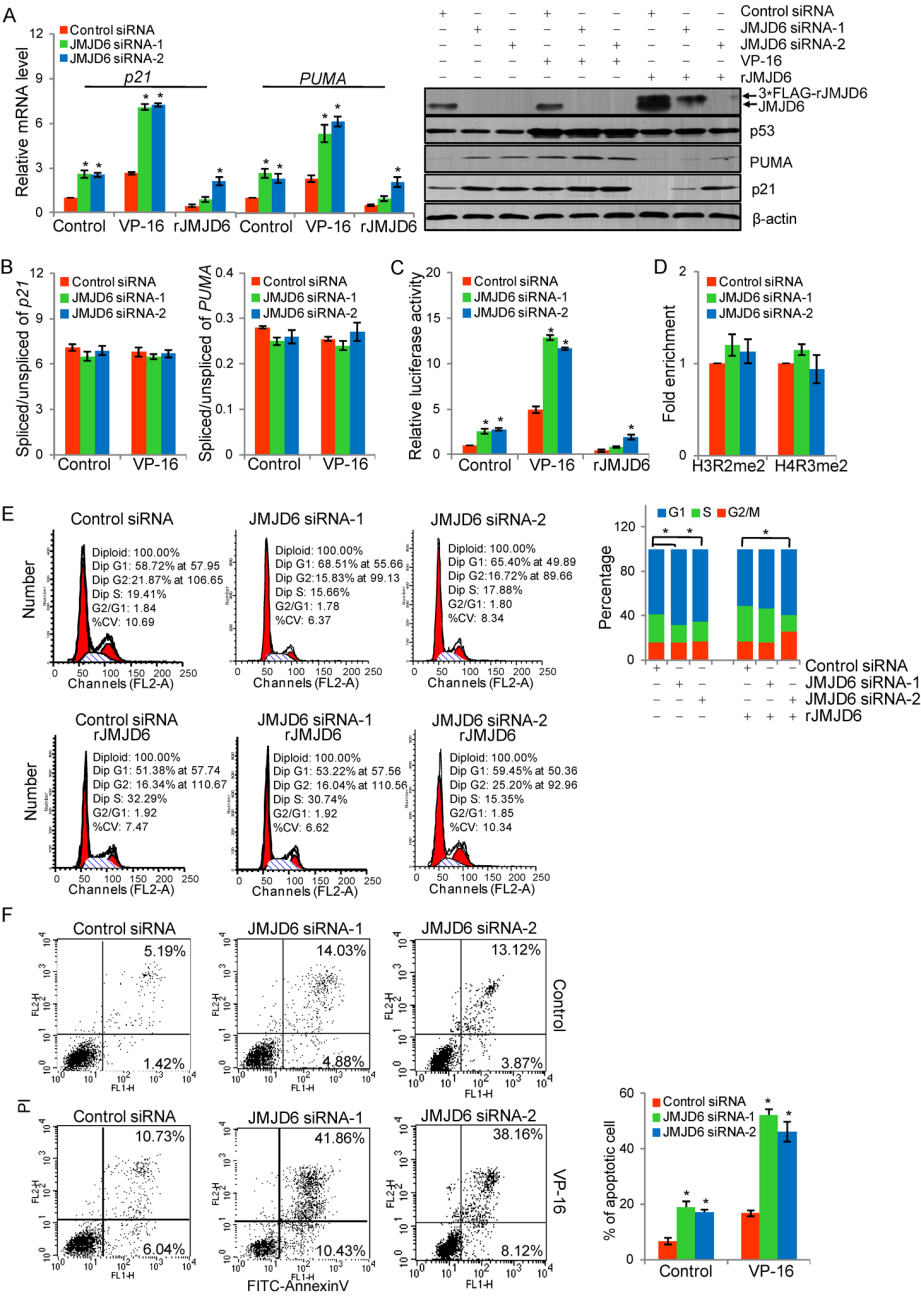
Negative regulation of p53 transcriptional activity by JMJD6. (A) Measurement of mRNA (left panel) and protein (right panel) levels of p21 and PUMA by real-time RT PCR and Western blotting in HCT116 cells that were transfected with JMJD6 siRNAs and/or JMJD6 siRNA-1–resistant JMJD6 form (rJMJD6) followed by treatment with or without VP-16. Each bar represents the mean ± S.D. for triplicate measurements. *p<0.05. (B) HCT116 cells were treated with control siRNA or JMJD6 siRNAs and challenged with or without VP-16. Real-time RT PCR was performed using exon–exon junction-specific or intron–exon junction-specific primers to measure spliced and unspliced mRNA levels of *p21* and *PUMA* by RT-qPCR analysis to determine splicing efficiency of *p21* (left panel) and *PUMA* mRNA (right panel). Each bar represents the mean ± S.D. for triplicate measurements. (C) Reporter assays in HCT116 cells that were transfected with JMJD6 siRNAs and/or rJMJD6 together with p21 promoter-driven luciferase reporter construct and challenged with or without VP-16. *p<0.05. (D) qChIP was performed in HCT116 cells treated with control siRNA or JMJD6 siRNA with indicated antibodies. (E) HCT116 cells transfected with JMJD6 siRNAs and/or rJMJD6 were synchronized by double thymidine block and released into the cell cycle. Cells were collected for cell cycle analysis by flow cytometry. Experiments were repeated three times and the data from a representative experiment are shown. *p<0.05. (F) HCT116 cells were transfected with control siRNA or JMJD6 siRNAs and challenged with or without VP-16 for 24 h. Annexin V/PI staining and flow cytometry were performed to assess the effect of JMJD6 on the apoptosis of HCT116 cells. Experiments were repeated three times, and the data from a representative experiment are shown. Each bar represents the mean ± S.D. for triplicate experiments. *p<0.05.

In order to investigate the biological significance of JMJD6-catalyzed p53 hydroxylation, we first examined the effect of JMJD6 on the p53 pathway. In these experiments, the expression of JMJD6 was knocked down in HCT116 cells by two specific siRNA and the cells were treated with or without etoposide phosphate (VP-16), an anticancer agent that inhibits topoisomerase II and induces DNA damages [48]. The expressions of *p21* and *PUMA*, two well-characterized p53 downstream target genes, were examined by real-time RT PCR and Western blotting. The results showed that although JMJD6 depletion did not affect the abundance of p53 in HCT116 cells, knockdown of JMJD6 resulted in increases in the levels of both mRNA (Figure 3A, left) and protein (Figure 3A, right) of p21 and PUMA in these cells. In addition, it appeared that JMJD6 affected the transcriptional activity of p53 under both normal and stressed conditions, as loss-of-function of JMJD6 was associated with a similar, albeit to a different extent, pattern of altered expression of p53 target genes regardless of whether it was with or without VP-16 treatment (Figure 3A). The silencing specificity of JMJD6 siRNA was validated using a JMJD6 siRNA-1–resistant JMJD6 form (rJMJD6) that was generated by synonymous mutation. Upon transfection of rJMJD6, the expression of p21 and PUMA was restored in cells treated with JMJD6 siRNA-1, but not in cells treated with JMJD6 siRNA-2 (Figure 3A). The expression of additional p53 target genes including *MDM2*, *Bax*, *Gadd45*, and *p53AIP1* were examined in HCT116 cells by real-time RT PCR. The results showed that JMJD6 depletion led to increases in mRNA levels of all the tested genes (Figure S2).

To investigate whether JMJD6 impacts on the p53 pathway through affecting transcription or mRNA splicing [5], real-time RT PCR was performed using exon–exon junction-specific or intron–exon junction-specific primers to measure the relative levels of spliced and unspliced *p21* and *PUMA* mRNAs in HCT116 cells that were transfected with control siRNA or JMJD6 siRNA. The results showed that loss of JMJD6 did not affect the splicing efficiency of the *p21* and *PUMA* transcripts (Figure 3B), favoring the idea that JMJD6 down-regulates p53 pathway through affecting transcription but not mRNA splicing. Consistent with this proposition, reporter assays with *p21* promoter-driven luciferase in HCT116 cells indicated that, under both normal and stressed conditions, JMJD6 down-regulates the expression of the p53 target gene at the transcriptional level (Figure 3C). In addition, although JMJD6 was described as a histone demethylase for H3R2 and H4R3 [4],[46], measurements of the levels of dimethylated (me2) H3R2 and H4R3 at *p21* promoter in HCT116 cells by quantitative chromatin immunoprecipitation (qChIP) detected no significant changes in H3R2me2 and H4R3me2 after JMJD6 depletion (Figure 3D). The recruitment of JMJD6 and p53 on *p21* promoter was also tested by qChIP assays in HCT116 cells. The experiments showed that p53, rather than JMJD6, was recruited to the promoter region of *p21* gene (Figure S3, left panel). Sequential ChIP or ChIP/Re-ChIP was performed in HCT116 cells to examine if JMJD6 and p53 co-occupy *p21* promoter. Soluble chromatins were first immunoprecipitated with antibodies against p53, and the immunoprecipitates were subsequently re-immunoprecipitated with antibodies against JMJD6, and vice versa. The results showed that *p21* promoter that was immunoprecipitated with antibodies against p53 could not be re-immunoprecipitated with antibodies against JMJD6 (Figure S3, left panel). The same was true when the initial ChIP was done with antibodies against JMJD6, in that p21 promoter was undetectable in precipitates following Re-ChIP with antibodies against p53 (Figure S3, left panel). To validate our experimentation, the recruitment of histone arginine methyltransferase CARM1, a known p53 coactivator protein [49], on p53 target gene promoter was measured to serve as a positive control (Figure S3, right panel). These experiments indicate that JMJD6 is not recruited by p53 on target gene promoters, suggesting that the hydroxylation of p53 by JMJD6 occurs not on gene promoters and not in the context of chromatin. Taken together, these data indicate that JMJD6 negatively regulates p53 transcriptional activity, possibly through hydroxylation modification.

In order to determine whether the negative effect of JMJD6 on the activity of p53 extends to physiologically relevant responses, HCT116 cells treated with control siRNA or JMJD6 siRNAs were first synchronized by double thymidine block and released into the cell cycle. The effect of JMJD6 knockdown on cell cycle progression was analyzed by propidium iodide (PI) staining and flow cytometry. Compared to control, JMJD6 knockdown was associated with a decrease of the cell population in the S phase and an increase of the cell population in G_1_ (Figure 3E). This effect could be, at least in part, reversed by expression of rJMJD6 in JMJD6 siRNA-1–treated HCT116 cells, but not in JMJD6 siRNA-2–treated HCT116 cells.

Next, we examined the effect of JMJD6 on cell apoptosis. HCT116 cells treated with control siRNA or JMJD6 siRNAs were challenged with VP-16 and analyzed by annexin V/PI double staining. Flow cytometry revealed that knockdown of JMJD6 in HCT116 cells resulted in an increased number of apoptotic cells, and treatment with VP-16 exacerbated the situation with more cells undergoing apoptosis (Figure 3F), suggesting that JMJD6 knockdown promotes cell apoptosis and sensitizes cells to DNA damaging agents.

### The Negative Impact of JMJD6 on p53 Pathway Is Through Its Effect on p53 Protein

**Figure 4:**
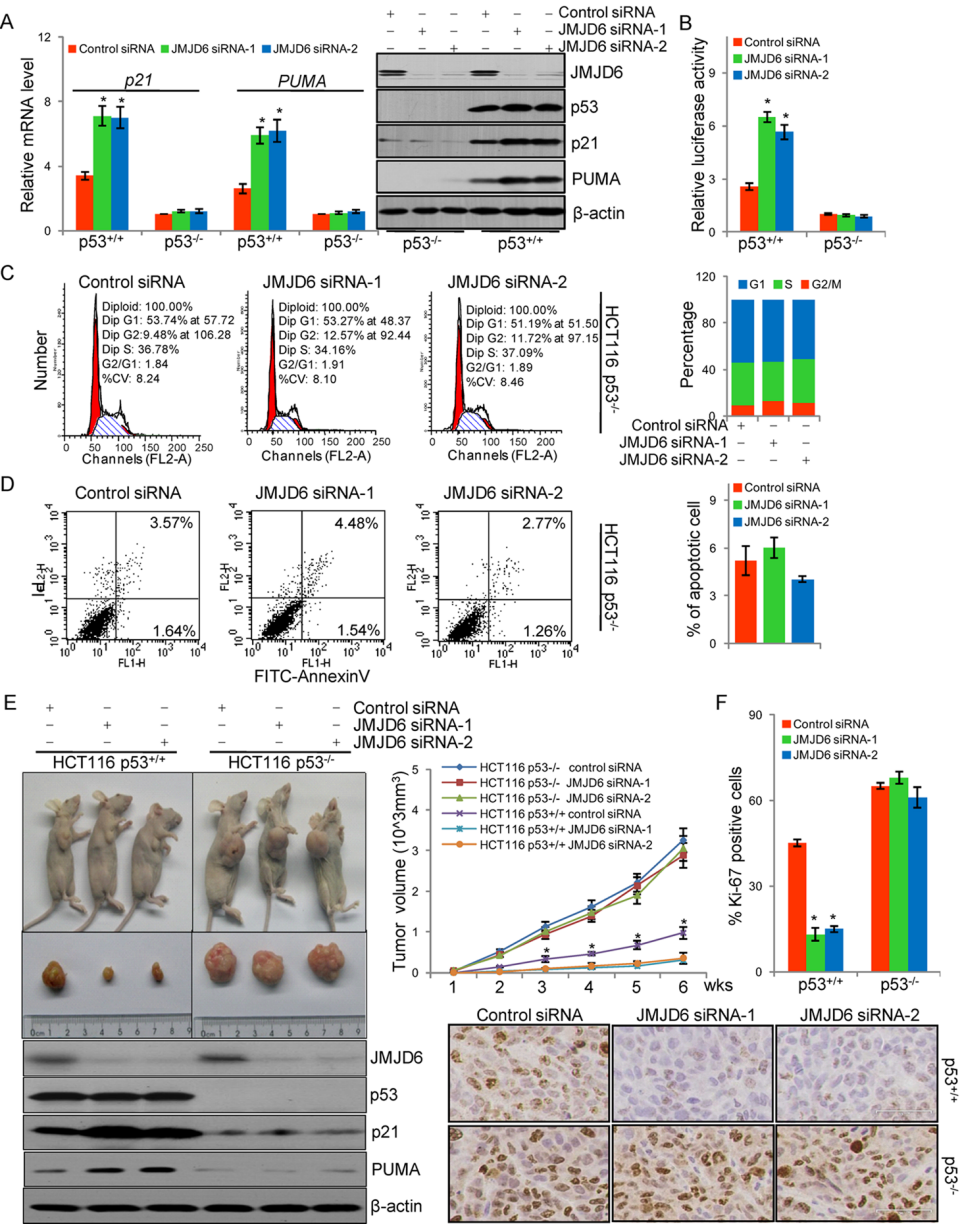
The negative impact of JMJD6 on p53 pathway is through its effect on p53 protein. (A) HCT116 p53^+/+^ or HCT116 p53^−/−^ cells were transfected with control siRNA or JMJD6 siRNAs. The mRNA levels of *p21* and *PUMA* were detected by real-time RT PCR and the levels of the indicated proteins were detected by Western blotting. (B) HCT116 p53^+/+^ or HCT116 p53^−/−^ cells were transfected with control siRNA or JMJD6 siRNAs together with p21 promoter-driven luciferase construct. Cells were then harvested and luciferase activity was measured and normalized to that of renilla. Each bar represents the mean ± S.D. for triplicate experiments. (C) HCT116 p53^−/−^ cells were transfected with control siRNA or JMJD6 siRNAs and were synchronized by double thymidine block and released into the cell cycle before cell cycle analysis by flow cytometry. Experiments were repeated three times and the data from a representative experiment are shown. (D) HCT116 p53^−/−^ cells were transfected with control siRNA or JMJD6 siRNAs, and annexin V/PI staining and flow cytometry were performed to assess cell apoptosis. (E) HCT116 p53^+/+^ or HCT116 p53^−/−^ cells were infected with lentivirus carrying a control siRNA or JMJD6 siRNAs, and were subcutaneously injected into the right anterior armpit of BALB/c nude mice. Tumors were measured weekly with Vernier calipers, and volume was calculated using the formula π/6×length×width^2^. Each point represents the mean ± S.D. for different animal measurements (*n* = 6). The levels of the indicated proteins extracted from xenograft tumor were detected by Western blotting. *p* values were determined by Student's *t* test. *p<0.01. (F) Immunohistochemical staining was performed in xenograft tumor sections using antibody against Ki-67. Scale bar, 36 µm. Proliferation was assessed by counting the number of Ki-67 positively stained nuclei and total number of cancer cells at 400× magnification in five representative regions of the tumor.

In order to gain further insights into the functional connection between JMJD6 and p53, the expression of the mRNA and protein of p21 and PUMA was measured in HCT116 p53^+/+^ or HCT116 p53^−/−^ cells. These experiments showed that JMJD6 depletion in HCT116 p53^+/+^ cells resulted in an elevated expression of mRNA and protein of p21 and PUMA (Figure 4A), whereas in HCT116 p53^−/−^ cells, knockdown of JMJD6 did not affect the expression of p21 and PUMA (Figure 4A). Reporter assays with p21 promoter-driven luciferase in HCT116 p53^+/+^ and HCT116 p53^−/−^ cells also indicated that the impact of JMJD6 depletion on the transcription of p21 was dependent on p53 (Figure 4B). Consistent with these results, in HCT116 p53^−/−^ cells that were treated with JMJD6 siRNA and/or transfected with wild-type p53 or p53K382R mutant expression plasmids, the mRNA expression of *p21* was elevated by both p53 and p53K382R, although the effect of p53K382R was slightly less pronounced than wild-type p53 (Figure S4A). JMJD6 knockdown led to an elevated induction of *p21* by wild-type p53 but had little effect on p53K382R transactivation activity (Figure S4A). These experiments suggest that the negative effect of JMJD6 on p53 transcriptional activity is dependent on hydroxylation of K382 of p53 by JMJD6. qChIP on promoters of six known p53 target genes (*p21*, *PUMA*, *MDM2*, *Bax*, *p53AIP1*, and *Gadd45*) in HCT116 p53^+/+^ or HCT116 p53^−/−^ cells under JMJD6 depletion was performed. The results showed that JMJD6 knockdown led to an increased recruitment of p53 on these gene promoters in HCT116 p53^+/+^, whereas it had little effect on p53 binding on these promoters in HCT116 p53^−/−^ cells (Figure S4B).

Next, we examined whether the effect of JMJD6 on cell cycle arrest is p53-dependent. For this purpose, HCT116 p53^−/−^ cells treated with control siRNA or JMJD6 siRNAs were synchronized by double thymidine block and released into the cell cycle. The effect of JMJD6 knockdown on cell cycle progression was analyzed by PI staining and flow cytometry. The results showed that, compared to HCT116 p53^+/+^ cells (Figure 3E), JMJD6 depletion was associated with no significant changes in the cell cycle profile in HCT116 p53^−/−^ cells (Figure 4C), suggesting that the effect of JMJD6 on cell cycle arrest is p53-dependent.

To test whether or not the effect of JMJD6 on cell apoptosis is p53-dependent, HCT116 p53^−/−^ cells were transfected with control siRNA or JMJD6 siRNAs. Annexin V/PI double staining and flow cytometry indicated that, compared to HCT116 p53^+/+^ cells (Figure 3F), HCT116 p53^−/−^ cells exhibited no differences in the population of apoptosis cells regardless of the treatment with control siRNA or JMJD6 siRNAs (Figure 4D), indicating that JMJD6-regulated cell apoptosis is dependent on p53 protein. Taken together, these data support the argument that JMJD6 negatively regulates the p53 pathway through acting on p53 protein.

To further explore the role of JMJD6 in p53-dependent cell proliferation and to investigate the possible role of JMJD6 in tumorigenesis *in vivo*, xenograft experiments were performed in nude mice by injecting HCT116 p53^+/+^ or HCT116 p53^−/−^ cells that were infected with lentivirus carrying either control siRNA or JMJD6 siRNA subcutaneously into the right anterior armpit of BALB/c nude mice. Measurements of the tumor growth over a period of 6 wk showed that all HCT116 p53^−/−^ xenografts grew significantly larger than HCT116 p53^+/+^ transplants, regardless of the infection of control siRNA or JMJD6 siRNAs (Figure 4E and Figure S8). However, the tumor growth was severely impaired in mice that received JMJD6-depleted HCT116 p53^+/+^ tumor transplants (Figure 4E). In agreement with the observation, immunohistochemical staining of Ki-67, a well-documented marker for cellular proliferation, in xenograft tumor section demonstrated that JMJD6-depleted HCT116 p53^+/+^ xenografts exhibited substantially fewer Ki-67–positive nuclei than HCT116 p53^+/+^ xenografts without JMJD6 depletion, whereas HCT116 p53^−/−^ xenografts displayed no difference in population of Ki-67–positive cells when cells were infected with either control siRNA or JMJD6 siRNAs (Figure 4F). These experiments indicate that JMJD6 play an important role in cell proliferation and tumorigenesis *in vivo*, and the data also support the notion that the effect of JMJD6 on cell proliferation and tumor development is dependent on p53.

### The Negative Effect of JMJD6 on p53 Pathway Depends on its Hydroxylase Activity

**Figure 5:**
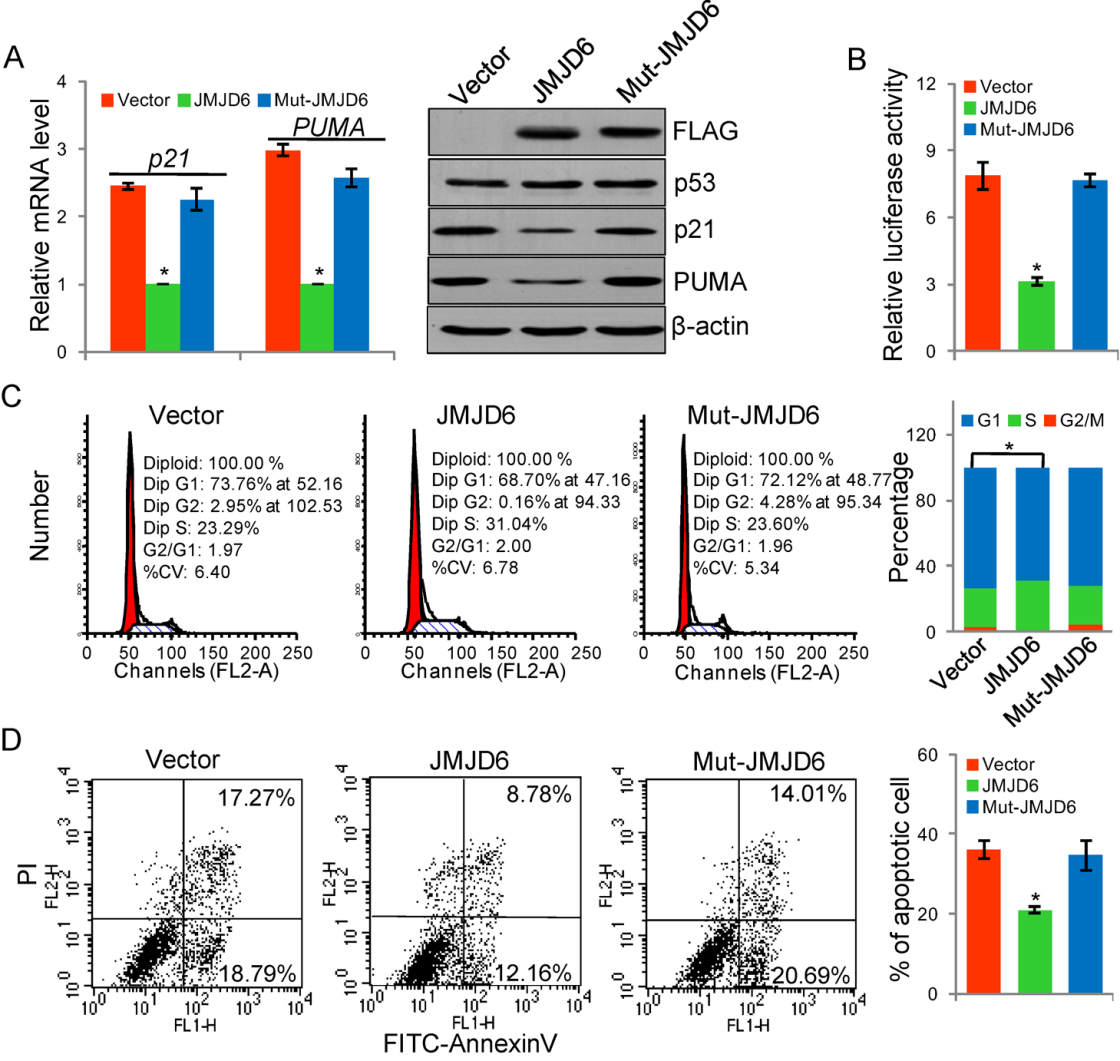
The negative effect of JMJD6 on p53 pathway depends on its hydroxylase activity. (A) HCT116 cells were transfected with vector, FLAG-JMJD6, or FLAG-JMJD6(H187A/D189A) (mut-JMJD6). The levels of the indicated proteins were detected by Western blotting. The mRNA levels of p21 and PUMA were detected by real-time RT PCR. (B) HCT116 cells were transfected with p21 promoter-driven luciferase construct together with vector, FLAG-JMJD6, or FLAG-JMJD6(H187A/D189A) (mut-JMJD6) plasmids. Cells were then harvested and luciferase activity was measured and normalized to that of renilla. Each bar represents the mean ± S.D. for triplicate experiments. (C) HCT116 cells transfected with vector or FLAG-JMJD6, or FLAG-JMJD6(H187A/D189A) (mut-JMJD6) were synchronized by double thymidine block and released into the cell cycle. Cells were collected for cell cycle analysis by flow cytometry. Experiments were repeated three times and the data from a representative experiment are shown. (D) HCT116 cells were transfected with vector, FLAG-JMJD6, or FLAG-JMJD6(H187A/D189A) (mut-JMJD6), and challenged with VP-16. Annexin V/PI staining and flow cytometry were performed to assess the effect of JMJD6 on the apoptosis of HCT116 cells.

Next, we tested whether or not the negative effect of JMJD6 on p53 pathway is through its hydroxylation of p53 protein. Analysis of the expression of p21 and PUMA by real-time RT PCR and Western blotting in HCT116 cells transfected with wild-type JMJD6 or JMJD6(H187A/D189A) indicated that overexpression of wild-type JMJD6 was associated with a significant decrease in the expression of mRNA and protein of p21 and PUMA, whereas transfection of JMJD6(H187A/D189A) was associated with little changes in the expression of p21 and PUMA (Figure 5A). Consistent with these results, reporter assays in HCT116 cells with *p21* promoter-driven luciferase also indicated that the catalytically inactive JMJD6(H187A/D189A) lost its effect on the transcription of p53 target genes (Figure 5B). Together with our observation that JMJD6 overexpression in HCT116 cells was associated with an increased p53 hydroxylation (Figure 2D), these experiments support an argument that JMJD6 negatively regulates the transcriptional activity of p53 through its hydroxylase activity.

In order to substantiate this argument, HCT116 cells were transfected with a vector, JMJD6, or JMJD6(H187A/D189A) expression plasmids. These cells were then synchronized by double thymidine block followed by release with fresh medium, and the effect of JMJD6 overexpression on cell cycle progression was analyzed by PI staining and flow cytometry. Compared to control, JMJD6 overexpression was associated with an increase (from 23.29% to 31.04%) of the cell population in the S phase and a decrease (from 73.76% to 68.70%) of the cell population in G_1_ (Figure 5C). The positive effect of JMJD6 on cell cycle progression was probably through its hydroxylase activity, as the catalytically defective JMJD6 mutant JMJD6(H187A/D189A) failed to promote cell cycle progression (Figure 5C). Analogously, annexin V/PI double staining and flow cytometry in HCT116 cells indicated that the inhibitory effect of JMJD6 on cell apoptosis was probably through its hydroxylase activity, as the catalytically defective JMJD6 mutant JMJD6(H187A/D189A) did not affect the number of cells that underwent apoptosis (Figure 5D). Taken together, if our interpretations are correct, these experiments indicate that the negative effect of JMJD6 on p53 pathway depends on its hydroxylase activity.

### The Interplay Between Hydroxylation and Acetylation of p53

**Figure 6:**
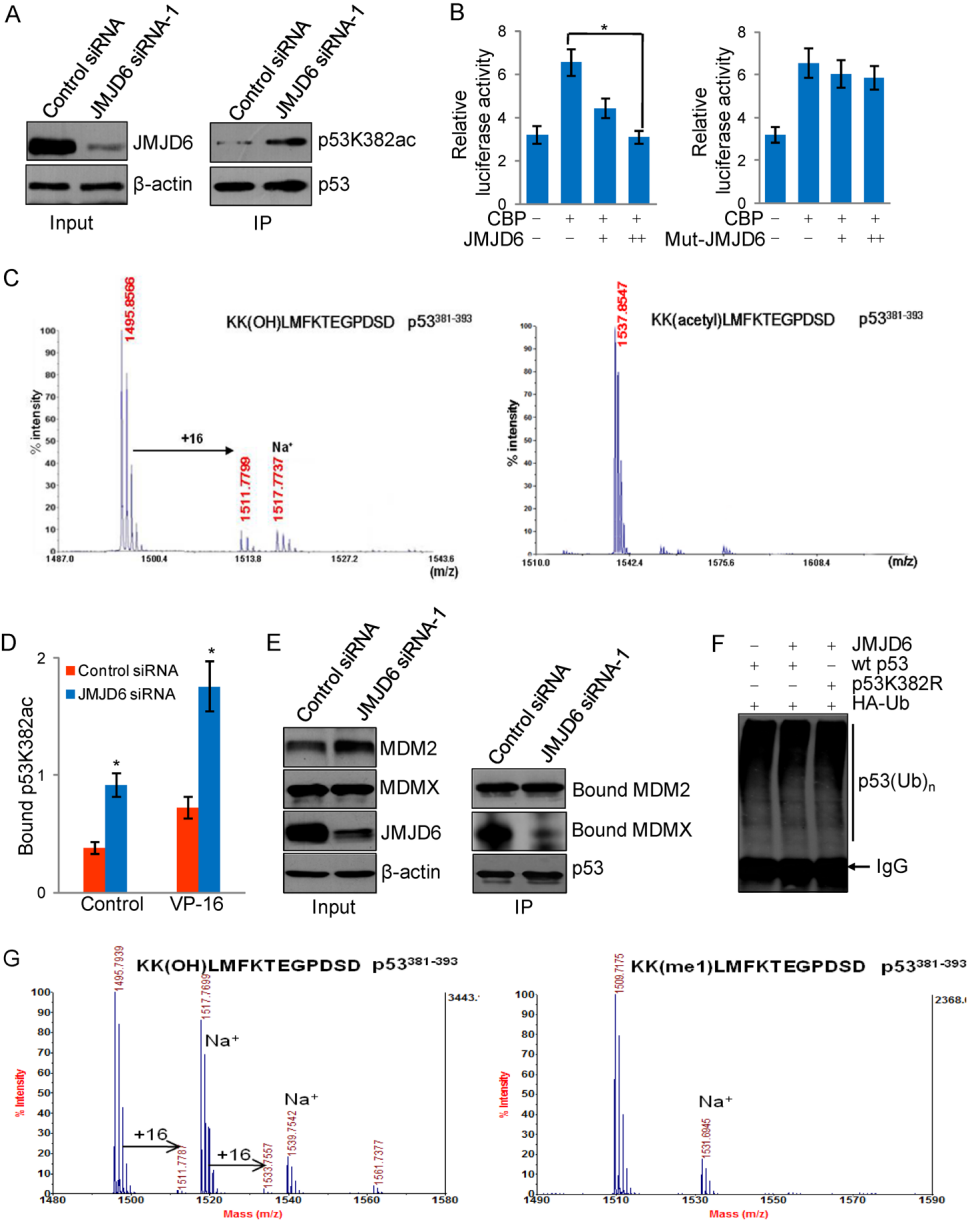
The interplay between hydroxylation and acetylation of p53. (A) Knockdown of JMJD6 promotes acetylation of p53^K382^. Lysates from HCT116 cells treated with control siRNA or JMJD6 siRNA-1 were immunoprecipitated with antibodies against p53 followed by immunoblotting with antibodies against acetyl-K382 of p53 (p53^K382ac^). (B) HCT116 cells were transfected with CBP and different amounts of FLAG-JMJD6 or FLAG-JMJD6(H187A/D189A) (mut-JMJD6) expression constructs together with p21 promoter-driven luciferase reporter. Cells were then harvested and luciferase activity was measured and normalized to that of renilla. Each bar represents the mean ± S.D. for triplicate experiments. (C) The peptide p53^381–393^ without (upper) or with (lower) K382 acetylation was incubated with recombinant JMJD6 in the presence of 2-OG and Fe(II). The mixture was then analyzed by MALDI/TOF. (D) qChIP assay was done to measure p53^K382ac^ bound to its response element and to a control site in p21 promoter in HCT116 cells treated with control siRNA or JMJD6 siRNA-1 and challenged with or without VP-16. (E) Lysates from HCT116 cells treated with control siRNA or JMJD6 siRNA-1 were immunoprecipitated with antibodies against p53 followed by immunoblotting with antibodies against MDMX or MDM2. (F) JMJD6 did not affect p53 ubiquitination *in vivo*. HCT116 p53^−/−^ cells were co-transfected with FLAG-JMJD6, HA-ubiquitin, wild-type p53, or p53 mutant (p53K382R). Forty-eight hours after transfection, cells were treated with MG132 for 6 h before cellular extracts were prepared for co-immunoprecipitation assays with anti-p53 followed by immunoblotting with anti-HA. (G) JMJD6 could not hydroxylate K382-methylated p53^381–393^ peptides. The peptide p53^381–393^ with or without K382 monomethylation was incubated with recombinant JMJD6 in the presence of α-ketoglutarate and Fe(II). The mixture was then analyzed by MALDI/TOF. (I) JMJD6 hydroxylates K382 of p53^381–393^ peptides; (II) JMJD6 could not hydroxylate K382-methylated p53^381–393^ peptides.

As stated above, most proteins including the p53 tumor suppressor are modified by a variety of enzymatic reactions, and it is currently believed that different modifications act either synergistically or antagonistically to define a final functional state for a particular protein. In effect, some of the modifications positively regulate protein activity, whereas others impact it negatively. In this regard, it is interesting to note that lysine 382 of p53 is also acetylated. As mentioned before, acetylation of K382 by CBP has been reported to enhance the transcriptional activity of p53 [37]. In light of our observations that JMJD6 and its catalyzed hydroxylation negatively regulate the transcriptional activity and cellular functions of p53, it is intriguing to speculate that the hydroxylation on K382 antagonizes the acetylation of this site. In order to test this hypothesis, HCT116 cells were treated with control siRNA or JMJD6 siRNA. p53 was immunoprecipitated from HCT116 cellular lysates with its specific antibodies, and the immunoprecipitates were then immunoblotted with an antibody against acetyl-K382 of p53 (p53^K382ac^). These experiments revealed a marked increase in the level of p53^K382ac^ in JMJD6-depleted cells (Figure 6A). Consistent with this, reporter assays in HCT116 cells with *p21* promoter-driven luciferase indicated that the enhancement effect of CBP on the reporter activity was abrogated in a dose-dependent fashion by wild-type JMJD6, but not JMJD6(H187A/D189A) (Figure 6B).

To investigate the relationship between hydroxylation and acetylation further, p53^381–393^ peptide with or without acetylation of lysine 382 (K382ac) was synthesized and then incubated with JMJD6 in the presence of Fe(II) and 2-OG. MALDI-TOF analyses detected hydroxylation modification on K382 with unacetylated peptide, but not with acetylated peptide (Figure 6C and Figure S5A,B), supporting the proposition that p53 hydroxylation by JMJD6 antagonizes p53 acetylation.

It is believed that the transcriptional activation of p53 by acetylation could be the result of an enhanced sequence-specific DNA binding [37] and/or a disruption of p53's association with MDMX, a predominant p53 corepressor [39]. In order to further support an antagonistic relationship between acetylation and hydroxylation of p53, we first examined, by qChIP, the alteration of DNA binding by p53^K382ac^ in JMJD6-depleted HCT116 cells. Analysis of the binding of p53 in its canonical sequence on the *p21* promoter showed a significant increase in p53^K382ac^ binding on the *p21* promoter in JMJD6-depleted HCT116 cells under both normal and stressed states (Figure 6D), suggesting that JMJD6 and p53 hydroxylation negatively regulate the DNA binding of p53. We then treated HCT116 cells with control siRNA or JMJD6 siRNA. Cellular lysates were prepared and co-immunoprecipitation experiments were performed with antibodies against p53. The immunoprecipitates were then immunoblotted with antibodies against MDMX or MDM2. We found that, compared to control siRNA-treated cells, JMJD6-depleted cells exhibited a diminished binding of p53 with MDMX (Figure 6E), although the protein levels of both p53 and MDMX in these cells were comparable and interaction of p53 with MDM2 was not affected (Figure 6E). These experiments suggest that JMJD6 and its catalyzed p53 hydroxylation promote p53–MDMX interaction and support our argument that hydroxylation antagonizes acetylation of p53.

Given that p53K382 residue can also be ubiquitinated, we next investigate the relationship between ubiquitination and hydroxylation of p53. *In vivo* ubiquitination was performed in HCT116 p53^−/−^ cells co-transfected with FLAG-JMJD6, HA-ubiquitin, wild-type p53, or p53 mutant (p53K382R). Immunoprecipitation of the cellular lysates with anti-p53 and immunoblotting with anti-HA revealed that JMJD6 overexpression had no evident effect on the level of p53 ubiquitination regardless of p53 status (Figure 6F), consistent with the results that JMJD6 does not affect the p53 stability in Figures 3A, 4A, and 5A.

To investigate the relationship between hydroxylation and methylation, we synthesized p53^381–393^ peptides with or without monomethylation of lysine 382 (K382me1). These peptides were then incubated with JMJD6 in the presence of Fe(II) and α-ketoglutarate. MALDI-TOF analyses detected hydroxylation modification on K382 with unmethylated peptides, but not with methylated peptides (Figure 6G), suggesting that lysine methylation counteracts hydroxylation on p53 lysine 382 residue.

### JMJD6 Is a Potential Biomarker for Colon Cancer Aggressiveness

**Figure 7:**
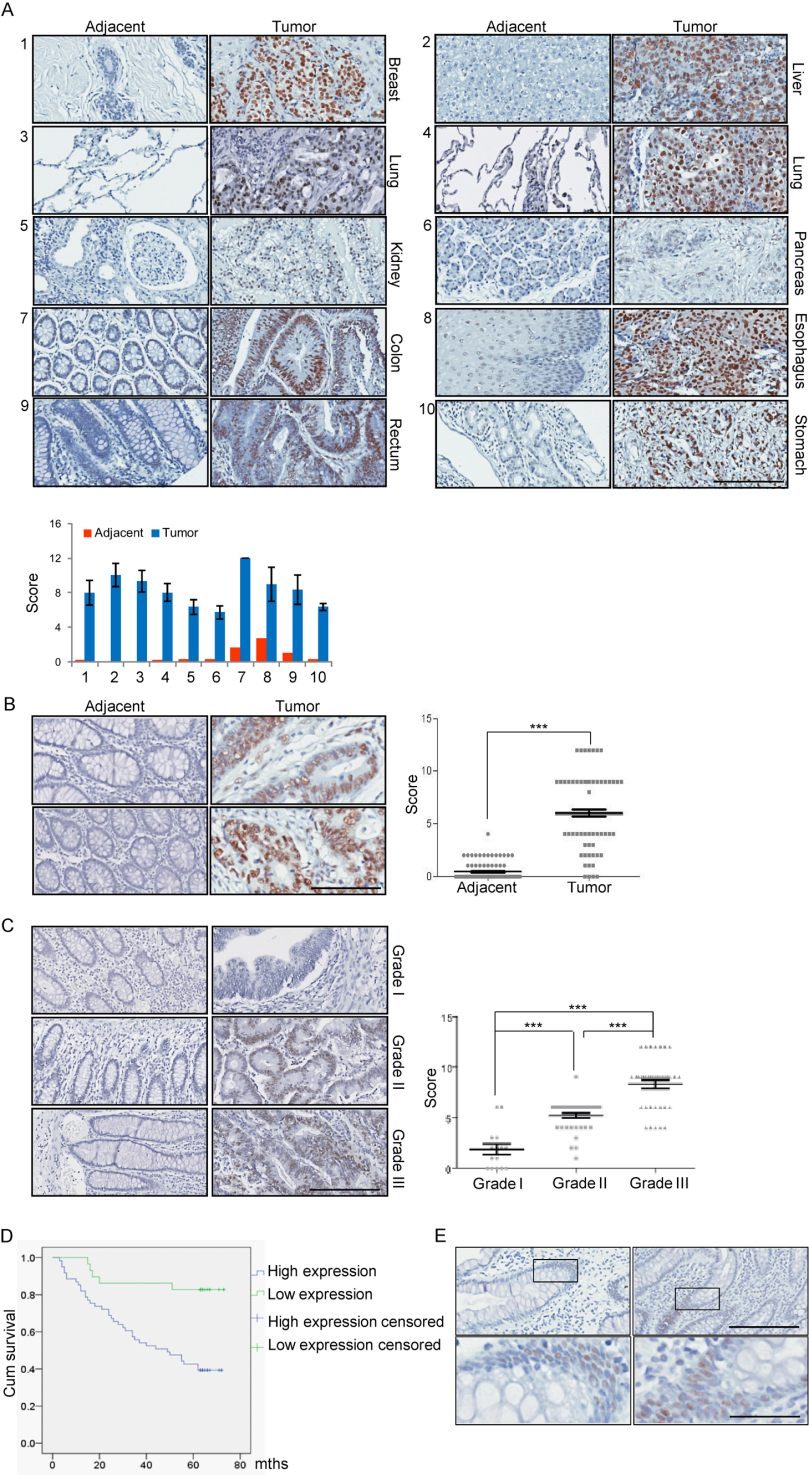
JMJD6 is a potential biomarker for colon cancer aggressiveness. (A) Immunohistochemical staining of JMJD6 in paired samples of breast ductal carcinoma (1), hepatocellular carcinoma (2), lung adenocarcinoma (3), lung squamous carcinoma (4), suprarenal epithelioma (5), pancreatic ductal carcinoma (6), colon adenocarcinoma (7), esophageal squamous carcinoma (8), rectal adenocarcinoma (9), and gastric adenocarcinoma (10) versus adjacent normal tissues. Representative tumor and adjacent normal sections stained with JMJD6 antibody are shown (magnification, ×25; scale bar, 200 µm). Each type of carcinoma included at least six paired samples and the scores were determined by evaluating the extent and intensity of immunopositivity. (B) Immunohistochemical staining of JMJD6 in 90 samples of colon adenocarcinomas paired with adjacent normal tissues. Representative sections from colon cancer (upper, tubular adenocarcinoma; lower, poorly differentiated adenocarcinoma) or adjacent normal tissue stained with JMJD6 antibody are shown (magnification, ×100; scale bar, 100 µm). The scores were determined by evaluating the extent and intensity of immunopositivity and were analyzed by paired-samples *t* test (***p<0.001). (C) Representative sections of histological grade I, II, and III of colon adenocarcinomas that were stained with JMJD6 antibody are presented (magnification, ×100; scale bar, 100 µm). The scores were determined by evaluating the extent and intensity of immunopositivity and were analyzed by two-tailed unpaired *t* test (***p<0.001). (D) Time-to-event data were plotted using Kaplan–Meier curves, and the 5-year survival rate of different groups was compared using the Mantel–Cox log-rank test (***p = 0.001). The *y*-axis represents the percentage of patients, and the *x*-axis represents the survival in months. (E) High expression of JMJD6 protein is found only in the base of intestinal glands (crypt of Lieberkuhn). Representative images of immunohistochemical staining of JMJD6 in normal intestinal glands are shown. Upper—magnification, ×25; scale bar, 200 µm. Lower—magnification, ×400; scale bar, 50 µm.

To investigate the tumorigenic effect of JMJD6 in a broad scope of cancers, we collected a series of carcinoma samples from breast, liver, lung, renal, pancreatic, colon, esophageal, rectal, and gastric cancer patients, with each type of carcinomas having at least six samples paired with adjacent normal tissues. Tissue microarray analysis by immunohistochemical staining showed that JMJD6 protein was mainly detected in the nuclei of cancer cells, and that its expression is up-regulated in all of the nine types of carcinomas, with higher levels in lung squamous carcinomas, lung adenocarcinomas, breast ductal carcinomas, and rectal adenocarcinomas, and the highest level in colon adenocarcinomas (Figure 7A and Figure S9).

To further explore the role of JMJD6 in colon carcinogenesis, we collected 90 colon carcinoma samples with paired adjacent normal tissues and performed tissue microarray analysis. Immunohistochemical staining showed that compared to normal tissues, JMJD6 protein was significantly up-regulated in colon adenocarcinomas (paired-samples *t* test, *p* = 0.001) (Figure 7B). Interestingly, there were significant positive correlations between JMJD6 expression and depth of invasion (*p*<0.05), lymph node metastasis (*p*<0.05), and advanced tumor node metastasis (TNM) stage (*p*<0.01) (Table S4). Moreover, higher JMJD6 protein appeared to be associated with poorer differentiation, as JMJD6 expression gradually increased with histological grade elevation (two-tailed unpaired *t* test, *p*<0.001) (Figure 7C). Remarkably, follow-up data showed that the survival rate of patients with high expression of JMJD6 was significantly lower than that with low expression of JMJD6 (log-rank test, *p* = 0.001) (Figure 7D). Although JMJD6 expression was statistically not an independent risk factor for short survival time (95% CI, 0.862 to 3.272; *p* = 0.127), Cox regression analysis indicated that high JMJD6 expression is associated with a shorter survival time with a hazard ratio of 1.680 compared to a low JMJD6 expression (Table S5), suggesting that JMJD6 is of clinical significance in the diagnosis, staging, and prognosis of patients with colon adenocarcinomas. Moreover, immunohistochemical staining of JMJD6 in normal tissue sections showed that high expression of JMJD6 is seen only in the base of intestinal glands (crypt of Lieberkuhn) where intestinal epithelia are mitotically active and undergo constant renewal (Figure 7E), suggesting that JMJD6 might be actively involved in proliferation and differentiation of intestinal cells. Together, these results indicated a robust association between JMJD6 expression and the aggressive clinical behaviors of colon adenocarcinomas, reflecting an underlying functional contribution of JMJD6 in colon carcinogenesis and providing a molecular basis for JMJD6 as a potential target for colon cancer therapy.

## Discussion

The sophisticated regulation of p53 is believed to be accomplished primarily through two mechanisms: regulation of the stability of p53 by a series of distinct E3 ligases [16],[50],[51] and modulation of the transcriptional activity of p53 via an assortment of posttranslational modifications. It has long been thought that the control of p53 turnover represents the leading component in the regulatory network of p53, and posttranslational modifications, on the other hand, mostly contribute to the finely tuned modulation of p53 transcriptional activity [27]. However, recent studies suggest that posttranslational modifications are as important, if not more so, as the regulation of p53 decay in the overall regulation of the p53 pathway [26],[52].

The p53 tumor suppressor is subjected to a myriad of posttranslational modifications, including, but probably not limited to, phosphorylation, acetylation, methylation, glycosylation, ubiquitination, neddylation, sumolyation, and poly-ribosylation [6]. The C-terminal domain of the p53 protein is subjected to a variety of modifications and thus is regarded as an important regulatory domain. For example, acetylation of lysine residues in the p53 C-terminal domain promotes p53 activation [37], whereas ubiquitination and methylation in this domain negatively regulate p53 transcriptional activity [18],[40],[41]. In the current report, we found that p53 is physically associated with Jumonji C domain-containing protein JMJD6 and is modified in its C-terminal domain via hydroxylation by JMJD6. Our data showed that hydroxylation of p53 in the C-terminal domain catalyzed by JMJD6 negatively regulates p53 activity and p53 hydroxylation occurs primarily on lysine 382 of p53.

Comparison of K382 sites of human p53 with the same or equivalent positions in p53 from other vertebrates indicated that the residue is evolutionarily conserved across different species, suggesting that the modification of p53K382 plays an important role in cellular function. Indeed, this site has been shown to be acetylated by CBP/p300 [37], ubiquitinated by MDM2 [16]–[20], and methylated by SET8 [41]. Interestingly, however, it was reported that homozygous p53^7KR^ mutated mice, in which seven C-terminal lysines including K382 of endogenous p53 are mutated (K to R), are viable and phenotypically normal [53]. In addition, in various cells derived from the p53^7KR^ mutated mice, the p53 protein has a normal half-life and only a slight impairment in transcriptional activity [53]. Therefore, whether or not cooperative or competitive mechanisms exist at the same site for these distinct modifications is currently not clear, largely due to mutations of the lysine 382 hindering acetylation, ubiquitination, as well as methylation, in addition to hydroxylation. At least in our study, knockdown of JMJD6 led to increased p53 acetylation, and JMJD6 could not hydroxylate K382-acetylated p53^381–393^ peptide, suggesting p53 hydroxylation by JMJD6 antagonizes p53 acetylation *in vivo*. In agreement with current understanding of p53 transcriptional regulation by acetylation, hydroxylation antagonizing acetylation and stabilizing the p53–MDMX interaction was associated with a decreased transcriptional activity of p53. It appears that hydroxylation has no evident effect on p53 ubiquitination, as our experiment showed that JMJD6 did not affect p53 ubiquitination and stability. We also noted that JMJD6 could not hydroxylate K382-methylated p53^381–393^ peptides, revealing that methylation and hydroxylation competes for p53 regulation. Although both have a negative outcome in terms of p53 activity, different modifications may result from different cellular signaling/stimuli, albeit the exact physiological conditions under which JMJD6 or SET8 become inactive are currently unknown.

Although subjecting the same lysine 382 of p53 to at least four different chemical modifications seems puzzling, it is conceivable that, considering the critical role of p53 in integrating cellular responses to various genotoxic and nongenotoxic stresses, the specific pattern of different modifications is likely to constitute a “p53 code” that reflects a particular environmental cue or cellular micromilieu. In this regard, it is of importance to note that acetylation, ubiquitination, methylation, and hydroxylation utilize, in addition to different enzymes, different cofactors and different auxiliary chemicals. It is also possible that these modifying enzymes are expressed or act optimally in different cell or tissue types. For example, the studies on SET8, SMYD2, and JMJD6 indicate that the expression of these enzymes exhibits a nonoverlapping tissue distribution pattern. Specifically, JMJD6 is highly expressed in thyroid and smooth muscle, whereas the highest expression of SET8 was found in the lymph node and the highest expression of SMYD2 was seen in retina and skeletal muscle cells, according to *Genecards* (www.genecards.org). Therefore, tissue-specific action of these different negative modifying enzymes on p53 is a distinct possibility. Clearly, future studies will be needed to fully understand the interplays and cross-talks among different posttranslational modifications of p53.

It was recently reported that JMJD6 is a top candidate gene robustly associated with poor differentiation and patient survival in breast cancer based on the analysis of an integrated “Super Cohort” (SC) of 15 individual Affymetrix array datasets comprising 2,116 breast cancer patients [54]. The same report also noted that JMJD6-regulated genes are enriched in cell-proliferation function by microarray analysis [54]. High expression of JMJD6 predicts unfavorable survival in lung adenocarcinoma [55]. Consistently, we detected overexpression of JMJD6 in carcinomas from multiple tissues. We found significant positive correlations between high JMJD6 expression and poor histological grade, advanced TNM stage, and shorter survival time in colon adenocarcinomas. Interestingly, immunostaining of JMJD6 in a normal tissue section showed that high expression of JMJD6 is detected only in the base of intestinal glands (crypt of Lieberkuhn) where intestinal epithelia are mitotically active and undergo constant renewal, with stem cells proliferating in the crypts, differentiating into five cell types of the intestinal epithelium, and migrating up the crypt–villus axis. Clearly, the functional importance of JMJD6 in carcinogenesis needs further investigation. Nonetheless, the information is consistent with our observations that JMJD6 is a negative regulator of p53 and an important regulator for cell proliferation and tumorigenesis, supporting the pursuit of JMJD6 as a novel biomarker of colon cancer progression and a potential target for colon cancer intervention.

A recent report showed the lysyl hydroxylation activity of JMJD6 towards histones [56], however it has been reported by another two independent labs that no evidence for hydroxylation of lysine residues (histones H2A, H2B, H3, and H4) was accrued in extensive MS-based analysis on endogenous histones [5],[57]. In our experiments, JMJD6-catalyzed lysyl hydroxylation in histone is also undetectable by MS. Reporter assays revealed that JMJD6 knockdown led to an elevated wild-type p53 transactivation activity, whereas it had little effect on p53K382R transactivation activity (Figure S6A). As a transient transfection experiment might not involve histones, these experiments suggest that the negative effect of JMJD6 on p53 transcriptional activity is not dependent on histone modification by JMJD6. In addition, examination of the recruitment of p53 proteins and the acetylation status of H4K5 and H4K8 on promoters of p53 target genes upon JMJD6 knockdown by quantitative ChIP (qChIP) showed that depletion of JMJD6 resulted in an increased binding of p53 protein on *p21* and *PUMA* promoters, which however was not concomitant with an increase in H4K5ac and H4K8ac (Figure S6B), arguing against the possibility that JMJD6 affects histone hydroxylation (Figure S7). Thus, to the best of our knowledge, the issue is still a subject of debate. Whether the functional diversity of JMJD6 represents different forms/modifications of JMJD6 itself in a particular cell type or reflects lineage-specific functionalities of JMJD6 in different cell types is currently unknown.

Future studies will be needed to investigate the cellular microenvironments and to determine the molecular mechanisms governing the functional specification of hydroxylation and methylation in the context of p53 repression. It will be interesting to gain more insights into the hydroxylation regulation, including the identification of a de-hydroxylation enzyme(s). It will also be interesting to explore the molecular mechanism underlying the functional diversity of JMJD6. In that sense, it is worthy of emphasizing that p53 is not the only substrate for JMJD6, as exemplified by U2AF65 hydroxylation by JMJD6; it is likely that future work will identify additional targets for JMJD6. Perhaps more relevant to our report, it is important to investigate the scope and the variety of the role of JMJD6 in tumorigenesis and to further consolidate the clinical significance of JMJD6 in future studies. Such efforts will provide a better understanding of the molecular activity and biological function of JMJD6 and will benefit the development of biomolecules for the diagnosis, prognosis, and treatment of cancers.

## Materials and Methods

### Ethics Statement

All studies related to animals were approved by the Animal Care Committee of Peking University Health Science Center. All studies concerning human tumor specimen were approved by the Ethics Committee of the Peking University Health Science Center, and informed consent was obtained from all patients.

### Plasmids, Antibodies, and Reagents

The cDNA for wild-type JMJD6 was amplified by PCR and ligated into *Xba* I/*EcoR* I sites of the pcDNA3.1 vector that contains one or three copies of FLAG. The GST-JMJD6 expression plasmid was constructed by cloning full-length JMJD6 into the pGEX-4T-3 vector. The JMJD6H187A/D189A mutant was generated by site-directed mutagenesis. The siRNA-resistant form of the JMJD6 construct (rJMJD6) was generated by synonymous mutation in which the sequence of JMJD6 cDNA at 674–692 bp, GA GGG AAC CAG CAA GAC GA, was substituted with GA GGA AAT CAA CAG GAT GA. All clones were confirmed by DNA sequencing. The sources of antibodies against the following proteins were as follows: FLAG (M2) and β-actin from Sigma, MDMX and acetyl-p53(p53^K382ac^) from Abcam, PUMA from Cell Signaling, p53 monoclonal antibody agarose conjugate p53 (FL-393) and JMJD6 from Santa Cruz, and p53 (DO-1) and p21 from MBL.

### Cell Culture and Transfection

HCT116 p53^+/+^ and HCT116 p53^−/−^ cells were from Dr. Yuxin Yin (Peking University Health Science Center) and maintained in Dulbecco's Modified Eagle's Medium (DMEM) (Hyclone) supplemented with 10% fetal bovine serum (FBS). All transfections were carried out using Lipofectamine 2000 (Invitrogen) according to the manufacturer's recommendations. Cells were transfected with siRNA oligonucleotides with Entranster-R (Engreen) transfection reagent. The sequences were as follows: JMJD6 siRNA-1, 5′-aaGAGGGAACCAGCAAGACGA-3′; JMJD6 siRNA-2, 5′-aaGUGUGGUGAGGAUAACGAU-3′.

### Immunopurification, Silver Staining, and Mass Spectrometry

HCT116 cells were transfected with FLAG-tagged JMJD6 expression plasmid. Forty-eight hours after transfection, cellular lysates were prepared by incubating the cells in lysis buffer. Anti-FLAG immunoaffinity columns were prepared using anti-FLAG M2 affinity gel following the manufacturer's suggestions. Cell lysates were obtained from about 5×10^8^ cells and applied to an equilibrated FLAG column of 1-ml bed volume to allow for adsorption of the protein complex to the column resin. After binding, the column was washed with cold PBS plus 0.1% Nonidet P-40. FLAG peptide was applied to the column to elute the FLAG protein complex as described by the vendor. Fractions of the bed volume were collected and resolved on SDS-polyacrylamide gel, silver stained, and subjected to LC-MS/MS (Agilent 6340) sequencing and data analysis. All LC-MS/MS analyses were performed on an Agilent 1100 Series HPLC-Chip/MS system interfaced to ion trap mass spectrometer (Agilent 6340). Peptides were separated by reversed-phase LC with a 40 nL enrichment column packed with ZORBAX 80 SB-C18, 5-µm particle size, and a 0.075×150 mm analytical column packed with ZORBAX 80 SB-C18, 5-µm particle size (HPLC-Chip cube; Agilent Technologies). Peptides were separated by using a gradient of 0%–40% buffer B (95% acetonitrile, 5% water, and 0.1% acetic acid) over 30 min followed by a gradient of 40%–95% buffer B over 10 min. The MS was operated in data-dependent mode to obtain a full MS spectrum followed by three MS/MS spectra obtained in the ion trap. Peptides selected for MS/MS interrogation were then placed on an exclusion list for 30 s to limit duplicate spectra. Protein identification was performed by using Spectrum Mill Proteomics Workbench Version A.03.03 (Agilent Technologies) and searching the human subset of the NCBI database. An initial search was performed by using two missed cleavages with complete proteolytic specificity, ±4 Da for the precursor mass, ±0.7 Da for the fragment masses, 40% minimum scored peak intensity, and 5+ for the maximum ambiguous charge state for the spectra with precursors of unassigned charge state. After this first search, a smaller database was created by using the proteins identified by these validated spectra. Further searches were performed against this database, allowing for up to four missed cleavages for spectra that contained a sequence tag >4.

### In-Gel Digestion

Immunoprecipitated p53 and recombinant GST-p53 were separated on SDS-PAGE, the gel was then stained with CBB, and the bands corresponding to the proteins of interest were excised and digested with trypsin protease (Promega) according to the standard protocol. The extracted peptides were used for mass spectrometry.

### GST Pull-Down Assay

GST pull-down assays were performed according to the procedure described previously [24],[44]. Briefly, equal amounts of GST fusion proteins (GST and GST-JMJD6) were immobilized on 50 µl of 50% glutathione-Sepharose 4B slurry beads (Amersham Biosciences) in 0.5 ml of GST pull-down binding buffer (10 mM HEPES, pH 7.6, 3 mM MgCl_2_, 100 mM KCl, 5 mM EDTA, 5% glycerol, 0.5% CA630). After incubation for 1 h at 4°C with rotation, beads were washed three times with GST pull-down binding buffer and resuspended in 0.5 ml of GST pull-down binding buffer before adding 5 µl of *in vitro* transcribed/translated p53 for 2 h at 4°C with rotation. The beads were then washed with 0.5 ml of ice-cold immunoprecipitation assay buffer and 1 ml of cold PBS. The bound proteins were eluted by boiling in 25 µl of loading buffer and resolved on SDS-PAGE.

### Co-Immunoprecipitation and Western Blotting

Co-immunoprecipitation and Western blotting were performed according to the procedure described previously [42],[58]. Briefly, HCT116 cellular lysates were prepared by incubating the cells in lysis buffer (50 mM Tris-HCl, pH 7.5, 150 mM NaCl, 0.5% NP-40, 2 mM EDTA) containing protease inhibitor cocktail for 20 min at 4°C, followed by centrifugation at 14,000 *g* for 15 min at 4°C. The protein concentration of the lysates was determined using the BCA protein assay kit according to the manufacturer's protocol (Pierce). For immunoprecipitation, 500 µl of protein was incubated with appropriate specific antibodies (1–2 g) for 12 h at 4°C with constant rotations; 60 µl of 50% protein A or G agarose beads was then added and the incubation was continued for an additional 2 h. Beads were then washed five times using the lysis buffer. Between washes, the beads were collected by centrifugation at 500 g for 5 min at 4°C. The precipitated proteins were eluted from the beads by resuspending the beads in 2× loading buffer and boiling for 5 min. The resultant materials from immunoprecipitation or cell lysates were resolved using 10% SDS-PAGE gels and transferred onto nitrocellulose membranes. For Western blotting, membranes were incubated with appropriate antibodies for 1 h at room temperature or overnight at 4°C followed by incubation with a secondary antibody. Immunoreactive bands were visualized using Western blotting Luminol reagent (Santa Cruz) according to the manufacturer's recommendation.

### Real-Time Reverse Transcription PCR

Total cellular RNAs were isolated with the TRIzol reagent (Invitrogen) and used for first strand cDNA synthesis with the Reverse Transcription System (Promega, A3500). Quantitation of all gene transcripts was done by qPCR using Power SYBR Green PCR Master Mix and an ABI PRISM 7300 sequence detection system (Applied Biosystems, Foster City, CA) with the expression of *GAPDH* as the internal control. The primer pairs used were as follows: *p21* forward primer, 5′-CATCCCGTGTTCTCCTTT-3′; *p21* reverse primer, 5′-GTGCCATCTGTT TACTTCTCA-3′; *PUMA* forward primer, 5′-AGACAAGAAGAGCAGCATCGACAC-3′; *PUMA* reverse primer, 5′-TAGGCACCTAGTTGGGCTCCATTT-3′; *JMJD6* forward primer, 5′-AAACTTTTGGAAGACTACAAGGTGC-3′; *JMJD6* reverse primer, 5′-CCCAGAGGGT CGATGTGAATC-3′; p53 forward primer, 5′-GTTCCGAGAGCTGAATGAGG-3′; p53 reverse primer, 5′-TCTGAGTCAGGCCCTTCTGT-3′; *GAPDH* forward primer, 5′-CCCACTCCTCC ACCTTTGAC-3′; *GAPDH* reverse primer, 5′-CATACCAGGAAATGAGCTTGACAA-3′; and *p21* intron forward primer, 5′-CCGAAGTCAGTTCCTTGTGG-3′; *p21* intron reverse primer, 5′-GGTCCCCTGTTGTCTGCC-3′.

### Reporter Assay

Luciferase activity was measured using a dual luciferase kit (Promega) according to the manufacturer's protocol. Each experiment was performed in triplicate and repeated at least three times.

### Hydroxylation Assay

GST-p53 (40 µM) or p53^382–393^ peptide was incubated with GST-JMJD6 (20 µM) in the presence of 2-OG (500 µM) and Fe(II) (400 µM) for 2 h at 37°C. The assay mixture was then separated on SDS-PAGE and the band corresponding to the molecular weight of GST-p53 was excised and digested with trypsin protease. The sample was analyzed using LC-MS/MS system (Agilent 6340) or MALDI-TOF-TOF 4800 Plus (ABI).

### qChIP

qChIP experiments were performed according to the procedure described previously [44],[59],[60]. The following primer pairs were used: *p21* promoter, 5′-AGACCCAGGCACAAACAT-3′ (forward) and 5′-GTCCATGTTACAGCCAGAC-3′ (reverse); *Gadd45* promoter, 5′-CTTCAGTGCATTAACCCTGG-3′ (forward) and 5′-CTTTAGCAGAGGCTAGAGGTG-3′ (reverse); *PUMA* promoter, 5′-GCGAGACTGTGGCCTTGTG-3′ (forward) and 5′-CGTTCCAGGGTCCACAAAGT-3′ (reverse); *p53AIP1* promoter, 5′-TGGGTAGGAGGTGATCTCACC-3′ (forward) and 5′- GAGCAGCACAAAATGGACTGGG-3′ (reverse); *MDM2* promoter, 5′-GGTTGACTCAGCTTTTCCTCTTG-3′ (forward) and 5′-GGCTATTTAAACCATGCATTTTCC-3′ (reverse); *Bax* promoter, 5′-TAATCCCAGCGCTTTGGAAG-3′ (forward) and 5′- TTGCTAGATCCAGGTCTCTGCA-3′ (reverse).

### Cell Flow Cytometry

HCT116 p53^+/+^ and HCT116 p53^−/−^ cells were synchronized by double thymidine block and released into the cell cycle. Cells were then trypsinized, washed with PBS, and fixed in 70% ethanol at 4°C overnight. After being washed with PBS, cells were incubated with RNAase A (Sigma) in PBS for 30 min at 37°C and then stained with 50 mg/ml PI. Cell cycle data were collected with FACSCalibur (Becton Dickinson) and analyzed with ModFit LT 3.0 (Verity Software House Inc., Topsham, ME).

### Tumor Xenografts

HCT116 p53^+/+^ or HCT116 p53^−/−^ cells were plated and infected with lentivirus carrying either control siRNA or JMJD6 siRNAs at MOI of 100. Forty-eight hours after infection, 5×10^6^ viable HCT116 p53^+/+^ or HCT116 p53^−/−^ cells in 200 µl PBS were injected into the right anterior armpit of 6- to 8-wk-old female BALB/c mice (Charles River, Beijing, China). Six animals per group were used in each experiment. Tumors were measured weekly using a Vernier calliper, and the volume was calculated according to the following formula: π/6×length×width^2^.

### Patients and Specimens

The samples of carcinomas and the adjacent normal tissues were obtained from surgical specimens from patients with breast, liver, lung, renal, pancreatic, colon, esophageal, rectal, or gastric cancer. Samples were selected from patients for whom complete information on clinicopathologic characteristics was available. The retrospective study for colon carcinoma consisted of 90 colon adenocarcinomas with paired adjacent normal tissues. Patients were diagnosed and treated from July 2006 to May 2007. The ages of the patients ranged from 24 to 90 y (median, 70 y; mean, 68.72 y). Of the patients, 46 were men and 44 were women. According to histological grading, 15 patients were at grade I, 37 were at grade II, and 38 were at grade III. According to the clinical TNM stage revised by the International Union against Cancer (UICC) in 2009, nine patients were stage I, 47 patients were stage II, 32 patients were stage III, and two patients were stage IV. All patients were followed up for survival. By August 2012 (the time of data analysis), 42 patients had died and 48 patients were alive. The median survival time was 63 mo.

### Tissue Microarray and Immunohistochemical Analysis

Tissue microarray blocks containing cores from cancer patients were constructed as described previously under institutional ethics committee approval with consent for the tissue microarray program (NUSIRB05-017) [61] and used for the analysis. Briefly, the antigen was retrieved by high pressure and incubation in 0.01 M sodium citrate buffer. Then the samples were blocked in 10% normal goat serum in PBS and incubated at 4°C overnight in primary antibody solution of anti-JMJD6 (1∶100). After being washed with 0.01 M PBS buffer, the samples were incubated with polymer HRP goat anti-mouse and rabbit IgG (GBI) for 30 min at room temperature, developed with DAB (3,3′-diaminobenzide tetrahydrochloride), and counterstained with hematoxylin (Zhongshan Golden Bridge Biotechnology Company). All specimens were examined by two pathologists who did not possess knowledge of the clinical data. In case of discrepancies, a final score was established by reassessment on a double-headed microscope. In scoring JMJD6 expression, both the extent and intensity of the immunopositivity were considered. The staining intensity was scored as follows: 0, negative; 1, weak; 2, moderate; 3, strong. The positivity was quantified according to the percentage of positive tumor cells: 0, <5%; 1, >5%–25%; 2, >25%–50%; 3, >50%–75%; 4, >75%. The final score was determined by multiplying the intensity and the quantity scores, which yielded a range from 0 to 12. The expression of JMJD6 was regarded as high expression when the score was >6.

### Statistical Analysis

Results are reported as mean ± S.D. unless otherwise noted. SPSS V.13.0 was used for statistical analysis. Comparisons between cancer and adjacent normal tissue were performed using paired-samples *t* test based on a bi-directional hypothesis for continuous variables. The chi-square test was used to examine the various clinicopathological characteristics of JMJD6 expression. The two-tailed unpaired *t* test was used to assess the relationship between JMJD6 and histological grades. Univariate survival analysis was conducted according to the Kaplan–Meier method, and the difference between the survival curves was analyzed with the log-rank test. Multivariate survival analysis was performed using the Cox proportional hazard model. Statistical significance was considered at a value of *p*<0.05.

## Supporting Information

Figure S1 **Wild-type JMJD6 hydroxylates p53^381–393^ at K382 of p53 in the presence of 2-OG, Fe(II)** ***in vitro*** **.** The peptides corresponding to amino acids 381–393 of p53 (wild-type p53, p53 K382A, or p53 K382R) were incubated with or without recombinant JMJD6 or JMJD6(H187A/D189A) in the presence of 2-OG and Fe(II) for 2 h at 37°C. The relevant ion fragments are labeled and the corresponding peptide positions are illustrated. (A) Experimental group with wt p53^381–393^, 2-OG, Fe(II), and wt JMJD6. (B) Experimental group with p53K382R, 2-OG, Fe(II), and JMJD6. (C) Experimental group with p53K382A, 2-OG, Fe(II), and JMJD6. (D) Experimental group with wt p53^381–393^, 2-OG, Fe(II), and JMJD6(H187A/D189A).
(TIF)

Figure S2 **The effect of JMJD6 on mRNA expressions of p53 target genes.** HCT116 cells were transfected with control siRNA or JMJD6 siRNAs. The mRNA levels of *MDM2*, *Bax*, *Gadd45*, and *p53AIP1* were detected by RT-qPCR. The results showed that JMJD6 depletion led to increases in mRNA levels of all the tested genes.
(TIF)

Figure S3 **JMJD6 is not recruited by p53 on target gene promoters.** ChIP and ChIP/Re-ChIP assays were performed with antibodies against the indicated proteins in HCT116 cells.
(TIF)

Figure S4 **The negative impact of JMJD6 on p53 transcription activity is through its effect on p53 protein.** (A) HCT116 p53^−/−^ cells were treated with JMJD6 siRNA and/or transfected with wild-type p53 or p53K382R mutant expression plasmids. The mRNA level of *p21* was detected by RT-qPCR. (B) The effect of JMJD6 on the p53 occupancy on promoters of p53 target genes (*p21*, *PUMA*, *MDM2*, *Bax*, *p53AIP1*, and *Gadd45*). HCT116 cells were treated with control siRNA or JMJD6 siRNA. Soluble chromatin was prepared and qChIP was performed with p53 antibody. Each bar represents the mean ± S.D. for triplicate experiments. *p* values were determined by Student's *t*-test; *p<0.05.
(TIF)

Figure S5 **JMJD6 cannot hydroxylate K382-acetylated p53^381–393^ peptide.** The peptide p53^381–393^ with or without K382 acetylation was incubated with recombinant JMJD6 in the presence of 2-OG and Fe(II) and then analyzed by MALDI/TOF. The relevant ion fragments are labeled and the corresponding peptide positions are illustrated. (A) K382 of p53^381–393^ peptide is hydroxylated by JMJD6. (B) K382-acetylated p53^381–393^ peptide is not hydroxylated by JMJD6. The relevant ion fragments are labeled and the corresponding peptide positions are illustrated.
(TIF)

Figure S6 **The negative effect of JMJD6 on p53 transcriptional activity is not dependent on histone modification by JMJD6.** (A) JMJD6 knockdown leads to an elevated wild-type p53 transactivation activity, whereas it has little effect on p53K382R transactivation activity. HCT116 p53^−/−^ cells treated with control siRNA or JMJD6 siRNA and/or vector, wild-type p53, and p53K382R mutant expression plasmids were transfected with a luciferase gene driven by *p21* promoter, *MDM2* promoter, or a synthetic promoter containing multiple p53 binding sites (pG13-Luc). Cells were then harvested and luciferase activity was measured and normalized to that of renilla. Each bar represents the mean ± S.D. for triplicate experiments. *p* values were determined by Student's *t* test; *p<0.05. (B) Depletion of JMJD6 results in an increased binding of p53 protein on *p21* and *PUMA* promoters, which is not concomitant with an increase in H4K5ac and H4K8ac. HCT116 cells were treated with control siRNA or JMJD6 siRNA. Soluble chromatin was prepared and qChIP was performed with the indicated antibodies. Each bar represents the mean ± S.D. for triplicate experiments. *p* values were determined by Student's *t* test; *p<0.05.
(TIF)

Figure S7 **The JMJD6 protein expression and the binding of p53 with JMJD6 under different stress conditions.** (A) HCT116 cells were treated with 1 µM adriamycin (Adr) for 6 h, 20 µM etoposide (VP-16) for 24 h, UV-C (60 J/m^2^), or were incubated under hypoxic (2% oxygen) conditions for 24 h. The protein expression was examined by Western blotting using antibodies against the indicated proteins. (B) The interaction between endogenous JMJD6 and p53 proteins were tested in HCT116 cells exposed to VP-16, or hypoxic condition. Cellular lysates were immunoprecipitated with anti-p53 (DO-1) followed by immunoblotting with JMJD6. Asterisk indicates nonspecific bands; arrow indicates JMJD6 bands.
(TIF)

Figure S8 **Original blot (left panel) and the repeated blot (right panel).** The proteins extracted from xenograft tumor were examined by Western blotting using antibodies against the indicated proteins.
(TIF)

Figure S9 **Immunohistochemical staining of JMJD6 in paired samples of breast ductal carcinoma versus adjacent normal tissues.** Representative tumor and adjacent normal sections stained with antibody diluent or JMJD6 antibody (1∶100 and 1∶300) are shown (magnification, ×25; scale bar, 200 µm).
(TIF)

Table S1 **Four potential JMJD6-interacting proteins were identified by LC-MS.**
(PDF)

Table S2 **JMJD6 hydroxylates p53 protein** ***in vitro*** **.** Recombinant p53 was incubated with or without recombinant JMJD6 in the presence or absence of α-ketoglutarate (2-OG) and Fe(II). The mixture was then separated on SDS-PAGE, and the band corresponding to the molecular weight of p53 was excised and digested with trypsin and analyzed by LC-MS/MS. The tables showed the theoretical m/z of “b” and “y” series of fragmented ions that were in agreement with the measured m/z. K, lysine; K-Hydroxylation, specifically hydroxylated lysine; M, methionine; M-Oxidation, random oxidized methionine. (A) Negative control group without Fe(II); (B) negative control group without 2-OG; (C) negative control group without JMJD6; (D) experimental group with 2-OG, Fe(II), and JMJD6.
(PDF)

Table S3 **Hydroxylation of p53 at K382** ***in vivo*** **.** Lysates from HCT116 cells were immunoprecipitated with anti-p53 monoclonal antibody-conjugated agarose. Bound proteins were eluted with p53 peptide, separated on SDS-PAGE, and analyzed by LC-MS/MS. The table showed the theoretical m/z of “b” and “y” series of fragmented ions that were in agreement with the measured m/z. Analysis by LC-MS/MS revealed the presence of modified p53^382–393^ peptide (M+2H)^2+^ containing hydroxylation K382.
(PDF)

Table S4 **Correlation between JMJD6 expression and clinicopathologic characteristics in colon adenocarcinomas by Chi-square test.**
(PDF)

Table S5 **Cox regression analysis of prognostic factors in colon adenocarcinomas.** B, partial regression coefficient; SE, standard error; *p*<0.05, statistically significant; HR, hazard ratio; CI, confidence interval.
(PDF)

## Abbreviations

ATM: ataxia telangiectasia mutated
ATR: ATM- and Rad3-related
CARM1: coactivator-associated arginine methyltransferase 1
CBB: Commassie brilliant blue
CBP: CREB binding protein
CHK1/2: checkpoint kinase 1/2
CPSF6: cleavage and polyadenylation specific factor 6
DNA-PK: DNA-dependent protein kinase
GST: glutathione S-transferase
2-OG: α-ketoglutarate
H3R2: histone H3 at arginine 2
H4R3: histone H4 at arginine 3
hnRNP A2/B1: heterogeneous nuclear ribonucleoprotein A2/B1
JFK: just one F-box and Kelch domain-containing protein
JMJD6: Jumonji domain-containing 6
LC-MS/MS: liquid chromatography-tandem mass spectrometry
MALDI-TOF: matrix-assisted laser desorption/ionization time-of-flight
qChIP: quantitative chromatin immunoprecipitation
rJMJD6: JMJD6 siRNA-1-resistant JMJD6 form
U2AF65: U2 small nuclear RNA auxiliary factor 2
VP-16: etoposide phosphate
