# Supplementary figures and images for: JMJD6 Promotes Colon Carcinogenesis through Negative Regulation of p53 by Hydroxylation
Source: PLoS Biol. 2014 Mar 25;12(3):e1001819. doi: 10.1371/journal.pbio.1001819 (PMC3965384; doi:10.1371/journal.pbio.1001819)

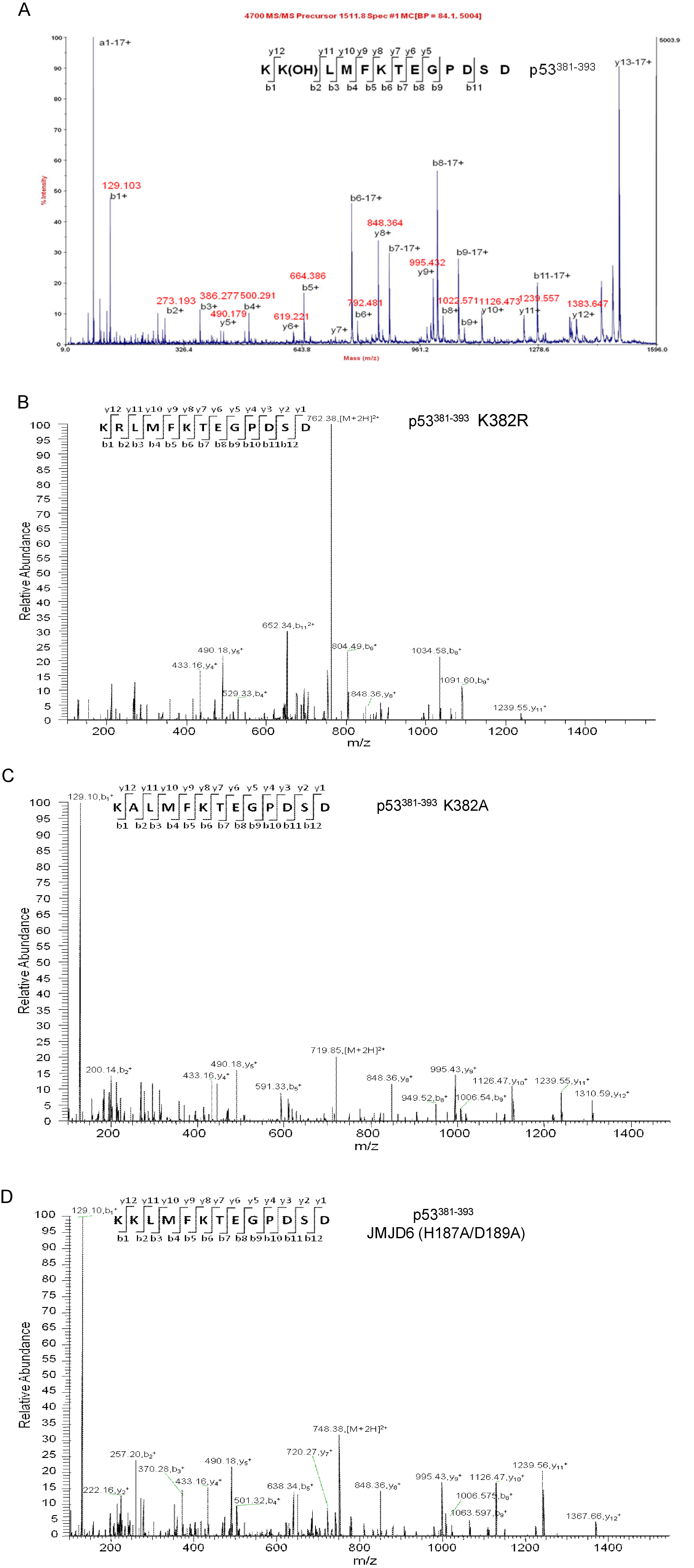

Supplement: Figure S1 — Wild-type JMJD6 hydroxylates p53381–393 at K382 of p53 in the presence of 2-OG, Fe(II) in vitro . The peptides corresponding to amino acids 381–393 of p53 (wild-type p53, p53 K382A, or p53 K382R) were incubated with or without recombinant JMJD6 or JMJD6(H187A/D189A) in the presence of 2-OG and Fe(II) for 2 h at 37°C. The relevant ion fragments are labeled and the corresponding peptide positions are illustrated. (A) Experimental group with wt p53381–393, 2-OG, Fe(II), and wt JMJD6. (B) Experimental group with p53K382R, 2-OG, Fe(II), and JMJD6. (C) Experimental group with p53K382A, 2-OG, Fe(II), and JMJD6. (D) Experimental group with wt p53381–393, 2-OG, Fe(II), and JMJD6(H187A/D189A). (TIF) [file pbio.1001819.s001.tif]

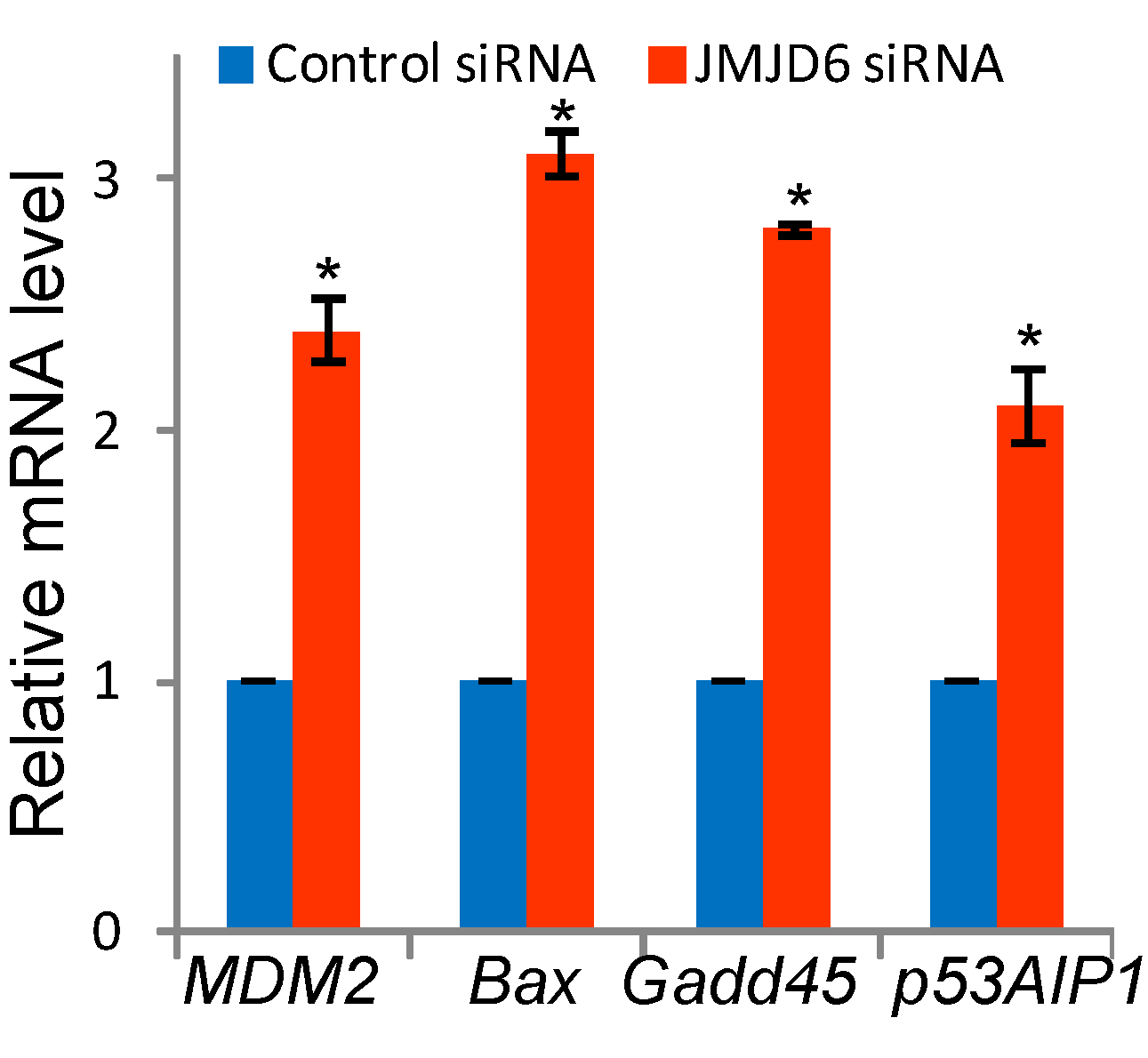

Supplement: Figure S2 — The effect of JMJD6 on mRNA expressions of p53 target genes. HCT116 cells were transfected with control siRNA or JMJD6 siRNAs. The mRNA levels of MDM2, Bax, Gadd45, and p53AIP1 were detected by RT-qPCR. The results showed that JMJD6 depletion led to increases in mRNA levels of all the tested genes. (TIF) [file pbio.1001819.s002.tif]

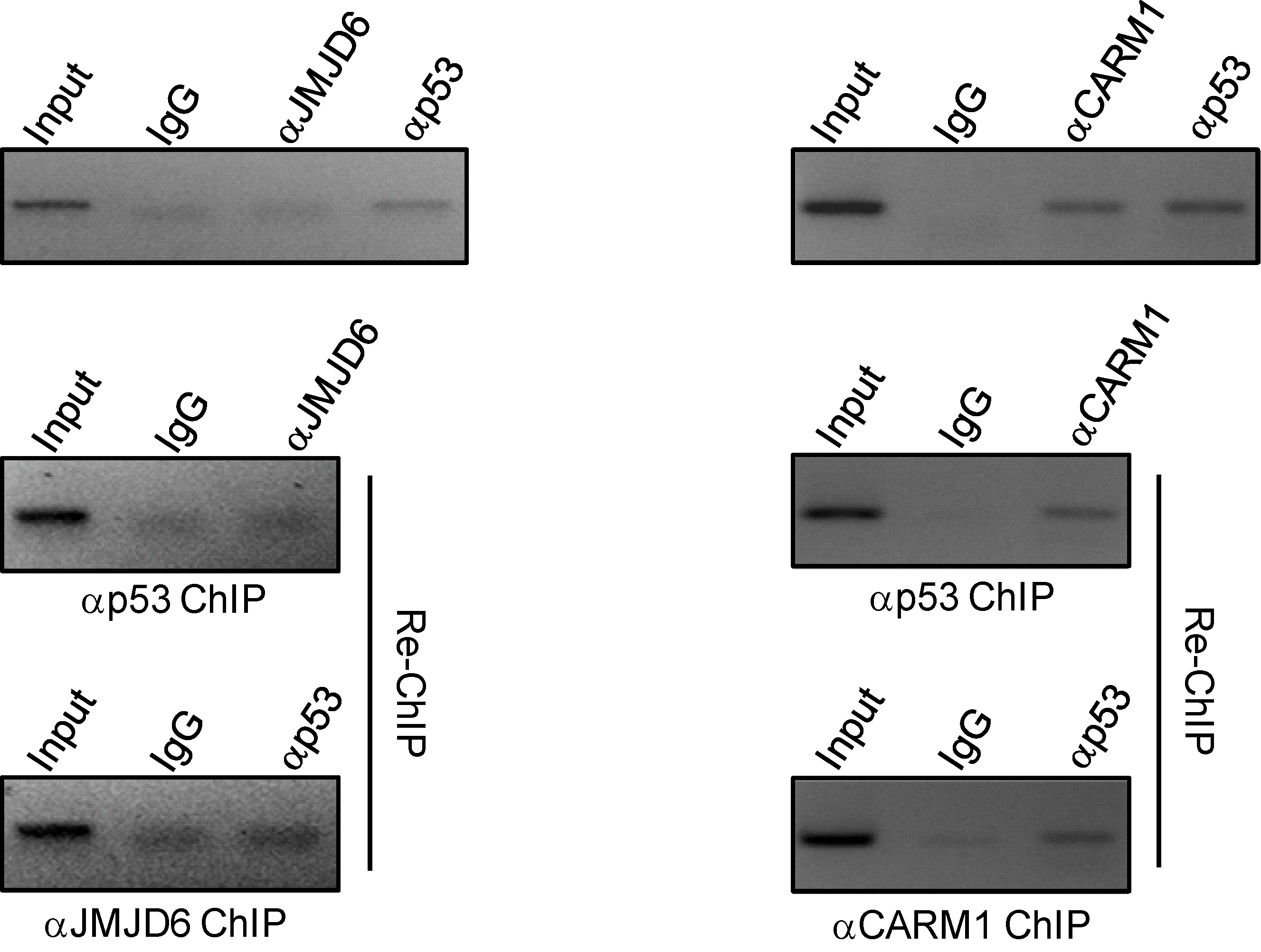

Supplement: Figure S3 — JMJD6 is not recruited by p53 on target gene promoters. ChIP and ChIP/Re-ChIP assays were performed with antibodies against the indicated proteins in HCT116 cells. (TIF) [file pbio.1001819.s003.tif]

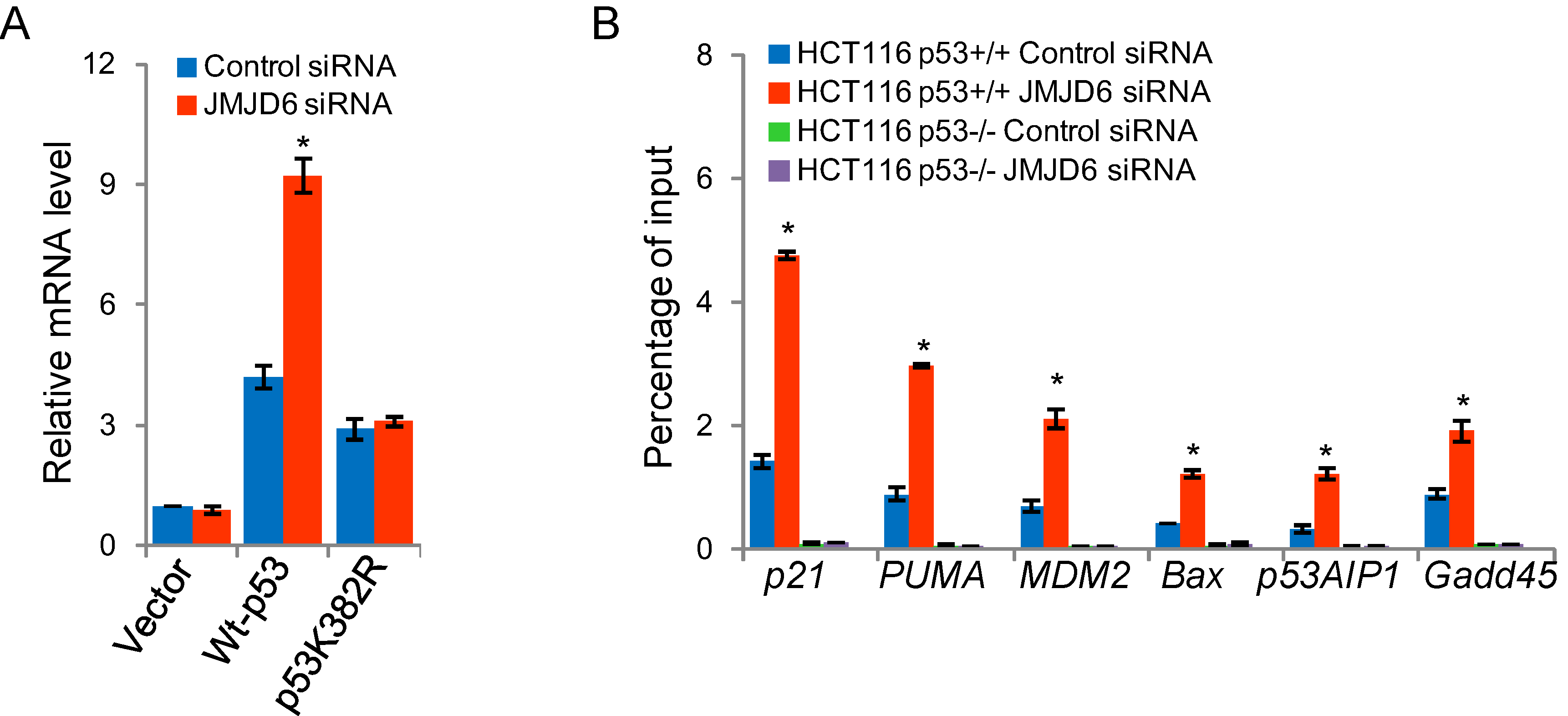

Supplement: Figure S4 — The negative impact of JMJD6 on p53 transcription activity is through its effect on p53 protein. (A) HCT116 p53−/− cells were treated with JMJD6 siRNA and/or transfected with wild-type p53 or p53K382R mutant expression plasmids. The mRNA level of p21 was detected by RT-qPCR. (B) The effect of JMJD6 on the p53 occupancy on promoters of p53 target genes (p21, PUMA, MDM2, Bax, p53AIP1, and Gadd45). HCT116 cells were treated with control siRNA or JMJD6 siRNA. Soluble chromatin was prepared and qChIP was performed with p53 antibody. Each bar represents the mean ± S.D. for triplicate experiments. p values were determined by Student's t-test; *p<0.05. (TIF) [file pbio.1001819.s004.tif]

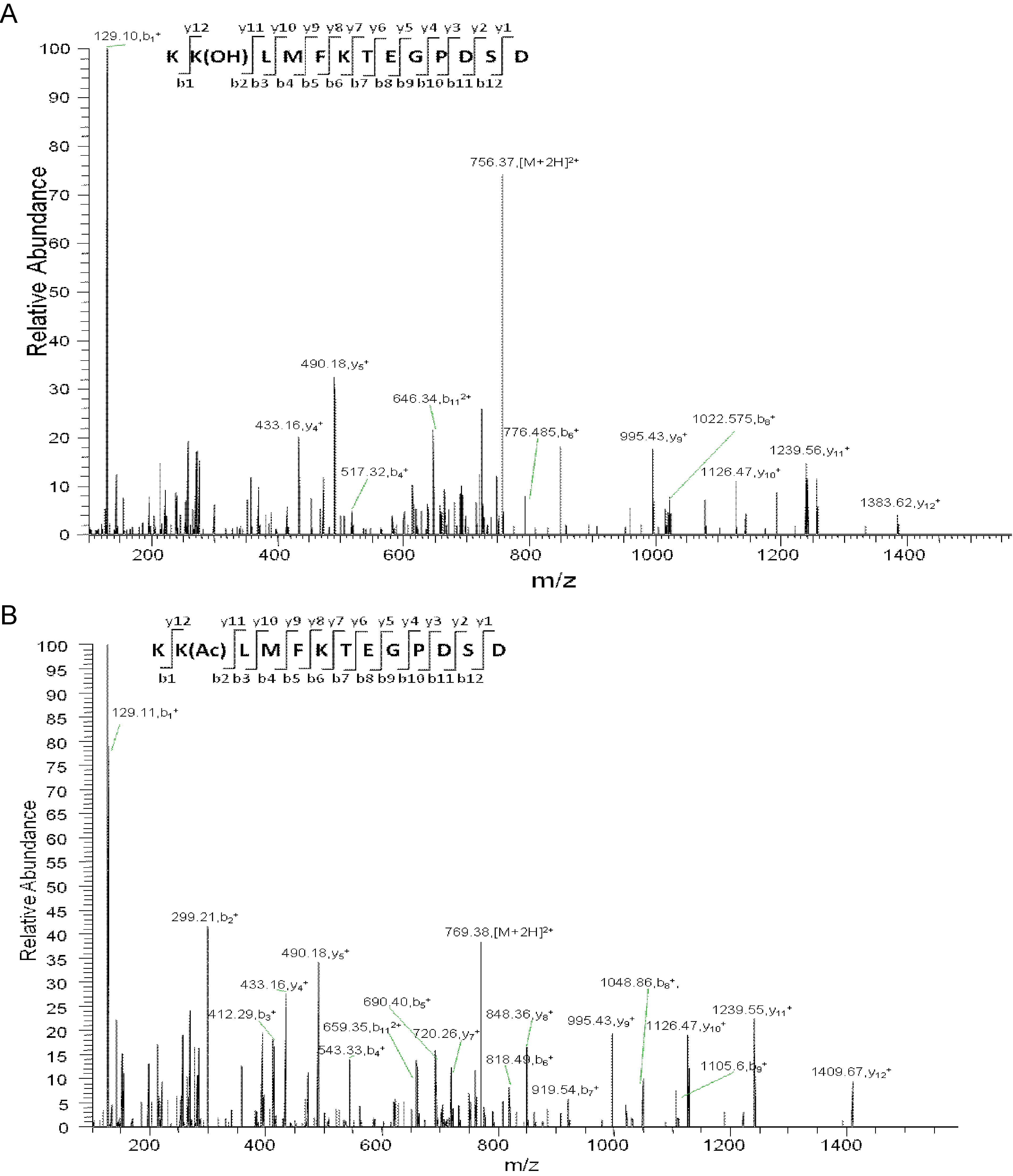

Supplement: Figure S5 — JMJD6 cannot hydroxylate K382-acetylated p53381–393 peptide. The peptide p53381–393 with or without K382 acetylation was incubated with recombinant JMJD6 in the presence of 2-OG and Fe(II) and then analyzed by MALDI/TOF. The relevant ion fragments are labeled and the corresponding peptide positions are illustrated. (A) K382 of p53381–393 peptide is hydroxylated by JMJD6. (B) K382-acetylated p53381–393 peptide is not hydroxylated by JMJD6. The relevant ion fragments are labeled and the corresponding peptide positions are illustrated. (TIF) [file pbio.1001819.s005.tif]

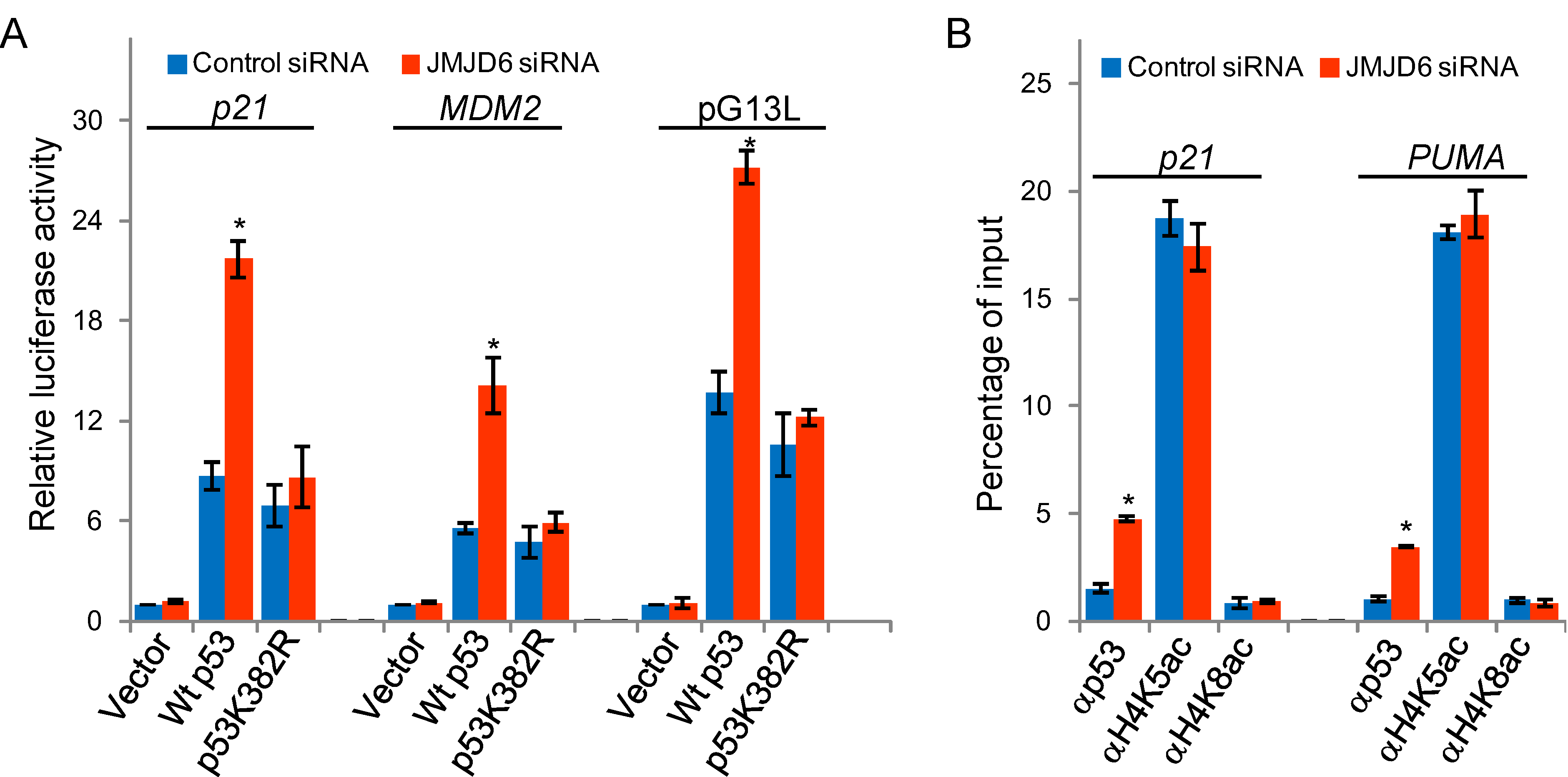

Supplement: Figure S6 — The negative effect of JMJD6 on p53 transcriptional activity is not dependent on histone modification by JMJD6. (A) JMJD6 knockdown leads to an elevated wild-type p53 transactivation activity, whereas it has little effect on p53K382R transactivation activity. HCT116 p53−/− cells treated with control siRNA or JMJD6 siRNA and/or vector, wild-type p53, and p53K382R mutant expression plasmids were transfected with a luciferase gene driven by p21 promoter, MDM2 promoter, or a synthetic promoter containing multiple p53 binding sites (pG13-Luc). Cells were then harvested and luciferase activity was measured and normalized to that of renilla. Each bar represents the mean ± S.D. for triplicate experiments. p values were determined by Student's t test; *p<0.05. (B) Depletion of JMJD6 results in an increased binding of p53 protein on p21 and PUMA promoters, which is not concomitant with an increase in H4K5ac and H4K8ac. HCT116 cells were treated with control siRNA or JMJD6 siRNA. Soluble chromatin was prepared and qChIP was performed with the indicated antibodies. Each bar represents the mean ± S.D. for triplicate experiments. p values were determined by Student's t test; *p<0.05. (TIF) [file pbio.1001819.s006.tif]

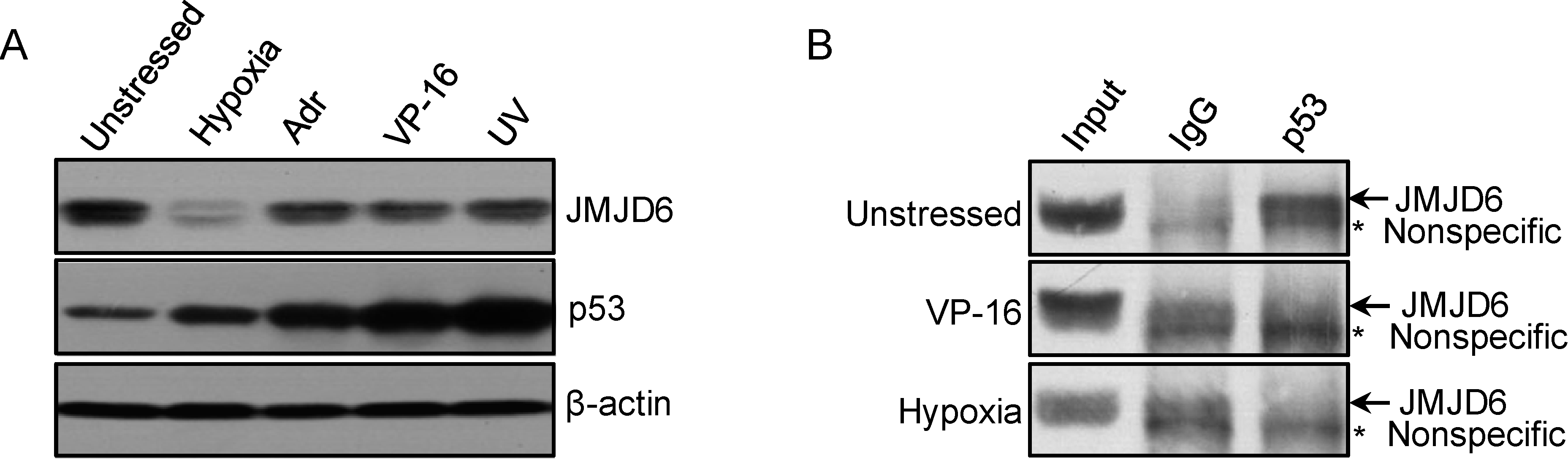

Supplement: Figure S7 — The JMJD6 protein expression and the binding of p53 with JMJD6 under different stress conditions. (A) HCT116 cells were treated with 1 µM adriamycin (Adr) for 6 h, 20 µM etoposide (VP-16) for 24 h, UV-C (60 J/m2), or were incubated under hypoxic (2% oxygen) conditions for 24 h. The protein expression was examined by Western blotting using antibodies against the indicated proteins. (B) The interaction between endogenous JMJD6 and p53 proteins were tested in HCT116 cells exposed to VP-16, or hypoxic condition. Cellular lysates were immunoprecipitated with anti-p53 (DO-1) followed by immunoblotting with JMJD6. Asterisk indicates nonspecific bands; arrow indicates JMJD6 bands. (TIF) [file pbio.1001819.s007.tif]

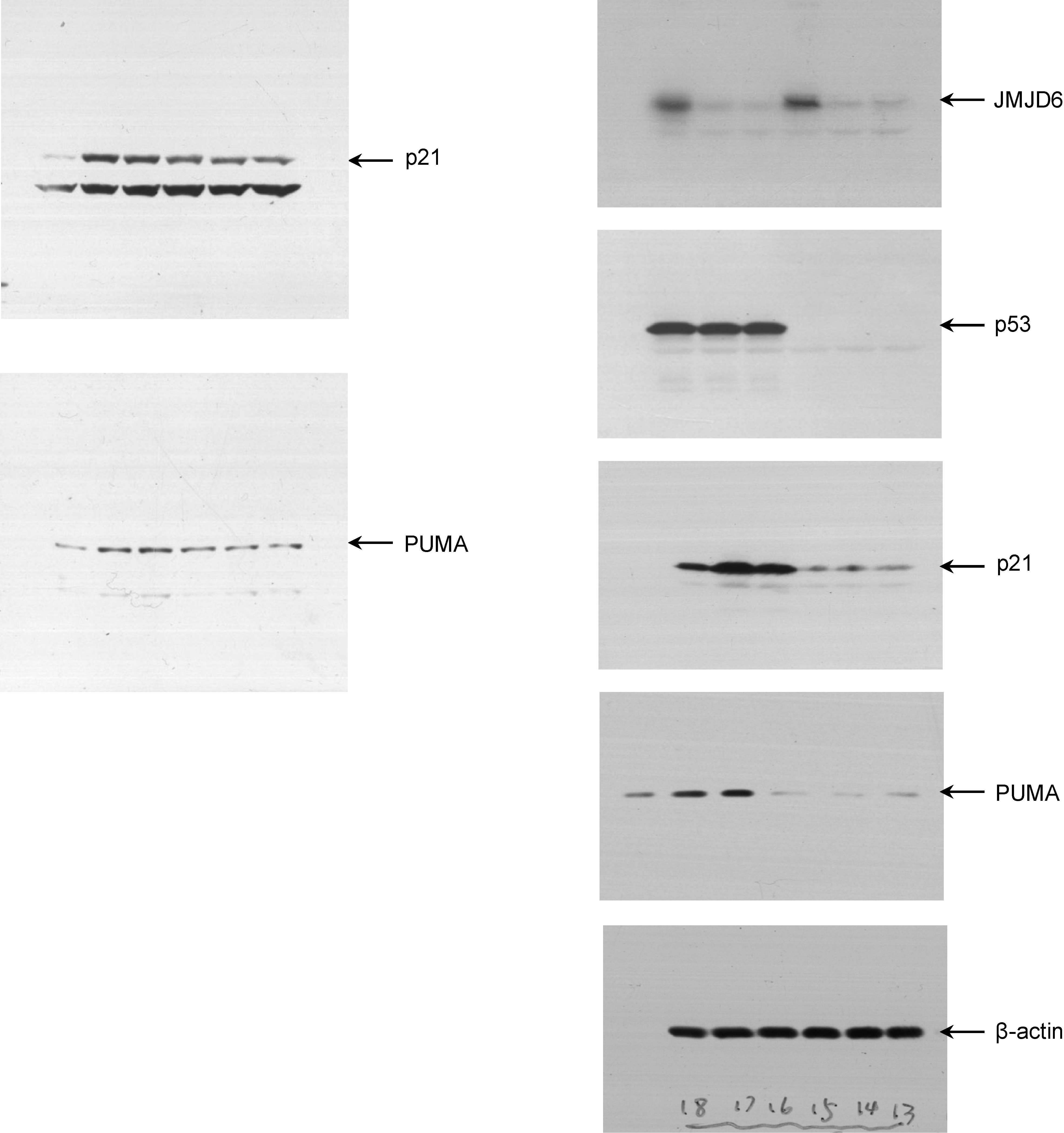

Supplement: Figure S8 — Original blot (left panel) and the repeated blot (right panel). The proteins extracted from xenograft tumor were examined by Western blotting using antibodies against the indicated proteins. (TIF) [file pbio.1001819.s008.tif]

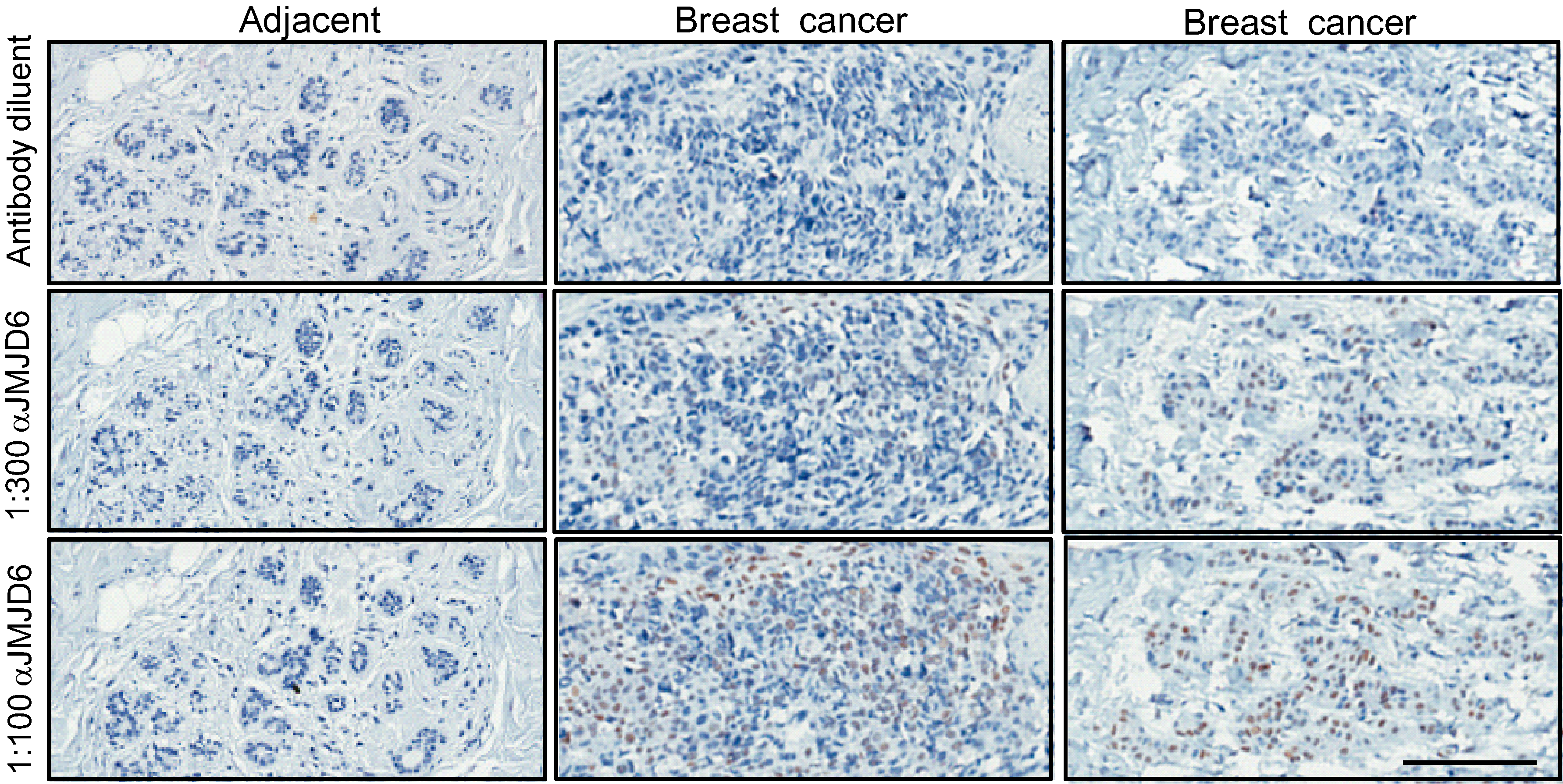

Supplement: Figure S9 — Immunohistochemical staining of JMJD6 in paired samples of breast ductal carcinoma versus adjacent normal tissues. Representative tumor and adjacent normal sections stained with antibody diluent or JMJD6 antibody (1∶100 and 1∶300) are shown (magnification, ×25; scale bar, 200 µm). (TIF) [file pbio.1001819.s009.tif]
