# Supplementary material for: JMJD6 Promotes Colon Carcinogenesis through Negative Regulation of p53 by Hydroxylation
Source: PLoS Biol. 2014 Mar 25;12(3):e1001819. doi: 10.1371/journal.pbio.1001819 (PMC3965384; doi:10.1371/journal.pbio.1001819)
Supplement: Table S1 — Four potential JMJD6-interacting proteins were identified by LC-MS. (PDF) [file pbio.1001819.s010.pdf]

Protein Name	Coverage	Peptide Sequence
p53	20%	(R)CPHHERCSDSDGLAPPQHLIR(V)<br>(R)RPILTIITLEDSSGNLLGR(N)<br>(R)LGFLHSGTAK(S)<br>(K)KGEPHHELPPGSTKR(A)<br>(K)KKPLDGEYFTLQIR(G)
CPSF6	14%	(R)AVSDASAGDYGSAIETLVTAISLIK(Q)<br>(R)TPLSEAEFEEIMNR(N)<br>(K)GFALVGVGSEASSK(K)<br>(R)AISSSAISR(A)<br>(K)GAAPNVVYTYTGKR(I)<br>(R)TPLSEAEREEIMNR(N)<br>(R)NRAISSSAISR(A)
hnRNP A2/B1	18%	(R)NMGGPYGGGNYGPGGSGGSGGYGGR(S)<br>(R)GGGGNFGPGPGSNFRGGSDGYGSGR(G)<br>(K)ALSRQEMQEVQSSR(S)
JMJD6	38%	(R)HNYYESFSLSPAAVADNVER(A)<br>(K)FFTDDLFQYAGEK(R)<br>(R)DEGGNQQDEAITWFNVIYPR(T)<br>(R)YERPYKPVVLLNAQEGWSAQEK(W)<br>(R)ADALQLSVEEFVER(Y)
Luc-like 2	9%	(K)SHLLDCCPHDILAGTR(M)<br>(R)VCEVCSAYLGLHDNDR(R)
