# Supplementary material for: JMJD6 Promotes Colon Carcinogenesis through Negative Regulation of p53 by Hydroxylation
Source: PLoS Biol. 2014 Mar 25;12(3):e1001819. doi: 10.1371/journal.pbio.1001819 (PMC3965384; doi:10.1371/journal.pbio.1001819)
Supplement: Table S2 — JMJD6 hydroxylates p53 protein in vitro . Recombinant p53 was incubated with or without recombinant JMJD6 in the presence or absence of α-ketoglutarate (2-OG) and Fe(II). The mixture was then separated on SDS-PAGE, and the band corresponding to the molecular weight of p53 was excised and digested with trypsin and analyzed by LC-MS/MS. The tables showed the theoretical m/z of “b” and “y” series of fragmented ions that were in agreement with the measured m/z. K, lysine; K-Hydroxylation, specifically hydroxylated lysine; M, methionine; M-Oxidation, random oxidized methionine. (A) Negative control group without Fe(II); (B) negative control group without 2-OG; (C) negative control group without JMJD6; (D) experimental group with 2-OG, Fe(II), and JMJD6. (PDF) [file pbio.1001819.s011.pdf]

A

bn	b^+	Seq.	y^+	yn
b1	129.1023	K	------	y12
b2	242.1863	L	1255.5512	y11
b3	389.2218	M-Oxidation	1142.4671	y10
b4	536.2902	F	995.4317	y9
b5	664.3852	K	848.3633	y8
b6	765.4328	T	720.2683	y7
b7	894.4754	E	619.2206	y6
b8	951.4969	G	490.1780	y5
b9	1048.5497	P	433.1565	y4
b10	1163.5766	D	336.1038	y3
b11	1250.6086	S	221.0768	y2
b12	-----	D	134.0448	y1

B

bn	b^+	Seq.	y^+	yn
b1	129.1023	K	-------	y12
b2	242.1863	L	1239.5562	y11
b3	373.2268	M	1126.4721	y10
b4	520.2952	F	995.4316	y9
b5	648.3902	K	848.3632	y8
b6	749.4378	T	720.2683	y7
b7	878.4804	E	619.2206	y6
b8	935.5019	G	490.1780	y5
b9	1032.5547	P	433.1565	y4
b10	1147.5816	D	336.1038	y3
b11	1234.6136	S	221.0768	y2
b12	---	D	134.0448	y1

C

bn	b^+	Seq.	y^+	yn
b1	129.1023	K	-------	y12
b2	242.1863	L	1239.5562	y11
b3	373.2268	M	1126.4721	y10
b4	520.2952	F	995.4316	y9
b5	648.3902	K	848.3632	y8
b6	749.4378	T	720.2683	y7
b7	878.4804	E	619.2206	y6
b8	935.5019	G	490.1780	y5
b9	1032.5547	P	433.1565	y4
b10	1147.5816	D	336.1038	y3
b11	1234.6136	S	221.0768	y2
b12	---	D	134.0448	y1

D

bn	b^+	Seq.	y^+	yn
b1	145.0972	K-Hydroxylation	---------	y12
b2	258.1812	L	1255.5512	y11
b3	405.2167	M-Oxidation	1142.4671	y10
b4	552.2851	F	995.4317	y9
b5	680.3801	K	848.3633	y8
b6	781.4277	T	720.2683	y7
b7	910.4703	E	619.2206	y6
b8	967.4918	G	490.1780	y5
b9	1064.5450	P	433.1565	y4
b10	1179.5720	D	336.1038	y3
b11	1266.6040	S	221.0768	y2
b12	------	D	134.0448	y1
