# Supplementary material for: JMJD6 Promotes Colon Carcinogenesis through Negative Regulation of p53 by Hydroxylation
Source: PLoS Biol. 2014 Mar 25;12(3):e1001819. doi: 10.1371/journal.pbio.1001819 (PMC3965384; doi:10.1371/journal.pbio.1001819)
Supplement: Table S3 — Hydroxylation of p53 at K382 in vivo . Lysates from HCT116 cells were immunoprecipitated with anti-p53 monoclonal antibody-conjugated agarose. Bound proteins were eluted with p53 peptide, separated on SDS-PAGE, and analyzed by LC-MS/MS. The table showed the theoretical m/z of “b” and “y” series of fragmented ions that were in agreement with the measured m/z. Analysis by LC-MS/MS revealed the presence of modified p53382–393 peptide (M+2H)2+ containing hydroxylation K382. (PDF) [file pbio.1001819.s012.pdf]

bn	b^+	Seq.	y^+	yn
b1	145.0972	K-Hydroxylation	---------	y12
b2	258.1812	L	1239.5560	y11
b3	389.2217	M	1126.4720	y10
b4	536.2901	F	995.4316	y9
b5	664.3851	K	848.3632	y8
b6	765.4327	T	720.2683	y7
b7	894.4753	E	619.2206	y6
b8	951.4968	G	490.1780	y5
b9	1048.5500	P	433.1565	y4
b10	1163.5770	D	336.1038	y3
b11	1250.6090	S	221.0768	y2
b12	---------	D	134.0448	y1
