# Supplementary material for: JMJD6 Promotes Colon Carcinogenesis through Negative Regulation of p53 by Hydroxylation
Source: PLoS Biol. 2014 Mar 25;12(3):e1001819. doi: 10.1371/journal.pbio.1001819 (PMC3965384; doi:10.1371/journal.pbio.1001819)
Supplement: Table S4 — Correlation between JMJD6 expression and clinicopathologic characteristics in colon adenocarcinomas by Chi-square test. (PDF) [file pbio.1001819.s013.pdf]

Variables	*n*	JMJD6 expression		*p* value
		Negative (*n*,%)	Positive (*n*,%)	
Histological grade				
I	15	13 (43.33%)	2 (3.33%)	*p*< 0.001
II	37	12 (40.00%)	25 (41.67%)	
III	38	5 (16.67%)	33 (55.00%)	
Gender				
Male	46	16 (53.33%)	30 (50.00%)	*P*=0.776
Female	44	14 (46.67%)	30 (50.00%)	
Age (years)				
>65	54	14(46.67%)	40(66.67%)	*p*=0.068
≤65	36	16 (53.33%)	20 (33.33%)	
Tumor size				
d<5	37	16 (53.33%)	21 (35.00%)	*p*=0.202
5≤d<10	42	12 (40.00%)	30 (50.00%)	
d≥10	11	2 (6.67%)	9 (15.00%)	
Depth of invasion				
T1	3	3 (10.00%)	0 (00.00%)	*p*=0.027
T2	7	4 (13.33%)	3 (5.00%)	
T3	69	21 (70.00%)	48 (80.00%)	
T4	11	2 (6.67%)	9 (15.00%)	
Lymph node				
N0	56	24 (80.00%)	32 (51.67%)	*p*=0.046
N1	25	4 (13.33%)	21 (35.00%)	
N2	9	2 (6.67%)	7 (11.67%)	
Distant metastasis				
M0	88	29 (96.67%)	59 (98.33%)	*p*=0.613
M1	2	1 (3.33%)	1 (1.67%)	
TNM stage				
I	9	7 (23.33%)	2 (3.33%)	*p*=0.009
II	47	15 (50.00%)	32 (53.34%)	
III-IV	34	8 (26.67%)	26 (43.33%)	
