# Supplementary material for: JMJD6 Promotes Colon Carcinogenesis through Negative Regulation of p53 by Hydroxylation
Source: PLoS Biol. 2014 Mar 25;12(3):e1001819. doi: 10.1371/journal.pbio.1001819 (PMC3965384; doi:10.1371/journal.pbio.1001819)
Supplement: Table S5 — Cox regression analysis of prognostic factors in colon adenocarcinomas. B, partial regression coefficient; SE, standard error; p<0.05, statistically significant; HR, hazard ratio; CI, confidence interval. (PDF) [file pbio.1001819.s014.pdf]

Prognostic variables	B	SE	Wald value	*p* value	HR	95% CI for HR	
						Lower	Upper
Poor histological grade	0.733	0.278	6.974	0.008	2.082	1.208	3.587
Lymph node metastasis	1.295	0.278	6.974	0.000	3.651	1.889	7.056
Depth of invasion	1.017	0.343	8.813	0.003	2.765	1.413	5.410
JMJD6 high expression	0.519	0.340	2.326	0.127	1.680	0.862	3.272
